# PINK1-parkin-mediated neuronal mitophagy deficiency in prion disease

**DOI:** 10.1038/s41419-022-04613-2

**Published:** 2022-02-18

**Authors:** Jie Li, Mengyu Lai, Xixi Zhang, Zhiping Li, Dongming Yang, Mengyang Zhao, Dongdong Wang, Zhixin Sun, Sharjeel Ehsan, Wen Li, Hongli Gao, Deming Zhao, Lifeng Yang

**Affiliations:** National Animal Transmissible Spongiform Encephalopathy Laboratory, College of Veterinary Medicine, China Agricultural University, Beijing, China

**Keywords:** Targeted gene repair, Target validation, Neurodegeneration, Neurodegeneration, Prion diseases

## Abstract

A persistent accumulation of damaged mitochondria is part of prion disease pathogenesis. Normally, damaged mitochondria are cleared via a major pathway that involves the E3 ubiquitin ligase parkin and PTEN-induced kinase 1 (PINK1) that together initiate mitophagy, recognize and eliminate damaged mitochondria. However, the precise mechanisms underlying mitophagy in prion disease remain largely unknown. Using prion disease cell models, we observed PINK1-parkin-mediated mitophagy deficiency in which parkin depletion aggravated blocked mitochondrial colocalization with LC3-II-labeled autophagosomes, and significantly increased mitochondrial protein levels, which led to inhibited mitophagy. Parkin overexpression directly induced LC3-II colocalization with mitochondria and alleviated defective mitophagy. Moreover, parkin-mediated mitophagy was dependent on PINK1, since PINK1 depletion blocked mitochondrial Parkin recruitment and reduced optineurin and LC3-II proteins levels, thus inhibiting mitophagy. PINK1 overexpression induced parkin recruitment to the mitochondria, which then stimulated mitophagy. In addition, overexpressed parkin and PINK1 also protected neurons from apoptosis. Furthermore, we found that supplementation with two mitophagy-inducing agents, nicotinamide mononucleotide (NMN) and urolithin A (UA), significantly stimulated PINK1-parkin-mediated mitophagy. However, compared with NMN, UA could not alleviate prion-induced mitochondrial fragmentation and dysfunction, and neuronal apoptosis. These findings show that PINK1-parkin-mediated mitophagy defects lead to an accumulation of damaged mitochondria, thus suggesting that interventions that stimulate mitophagy may be potential therapeutic targets for prion diseases.

## Introduction

Prion diseases are a group of chronic, fatal, neurodegenerative diseases that can infect humans and animals [1, 2]. The key event in the pathogenesis of Prion diseases is the conformational conversion of a normal cell surface glycoprotein (the cellular isoform of the prion protein, PrP^C^) into a pathogenic isoform (named PrP^Sc^) that is characterized by a high content of β-sheet structure [3]. Since the neurotoxicity of PrP106-126 was first reported in 1993 [4], numerous laboratories have used this peptide as an experimental model to investigate the molecular mechanisms of PrP^Sc^ neurotoxicity [5–10]. This peptide maintains most of the pathogenic characteristics of PrP^Sc^, including neurotoxicity, gliotrophic activity, proteinase-K resistance, and β-sheet structure. Moreover, this peptide was reported to induce apoptotic death in primary cultures of hippocampal, cortical, and cerebellar neurons [11]. PrP106-126 is proved useful in clarifying the structural and physicochemical features underlying PrP neurotoxicity [10, 11].

A persistent accumulation of damaged mitochondria in neurons has been associated with aging and neurodegenerative diseases, including prion diseases [12–17]. Mitophagy, one of the intracellular mitochondrial quality control pathways, can selectively remove damaged mitochondria, which may be associated with the accumulation of damaged mitochondria in prion diseases [18]. Many studies have reported the importance of PINK1-parkin dependent mitophagy pathways in neurons: PINK1-parkin forms a signal transduction pathway that labels damaged mitochondria with ubiquitin chains [19] and then by recruiting and binding mitophagy receptors [20]. Those receptors direct the autophagosome membrane to surround the ubiquitinated mitochondria, which is then eliminated through autophagosome and lysosome pathways [21–24].

There is evidence that altered PINK1-Parkin-related mitophagy may be involved in the pathogenesis of neurodegenerative diseases such as Alzheimer’s disease (AD) and Parkinson’s disease (PD) [12, 16, 25–27]. Therefore, this study aims to investigate the correlation between damaged mitochondrial accumulation and mitophagy in the prion disease cell model. In this study, we found that PINK1-Parkin-related mitophagy was impaired in the prion disease cell model induced by PrP106-126. Neurons with overexpressed parkin, PINK1, and added mitophagy activator NMN had alleviated mitophagy deficiency and protection from apoptosis. These findings showed that the accumulation of damaged mitochondria in the prion disease model is due to defective mitophagy. Therefore, PINK1-parkin-mediated mitophagy may be a potential therapeutic target for prion diseases.

## Results

### PrP106-126 induced mitophagy deficiency in mouse neuroblastoma N2a cells

**Fig. 1:**
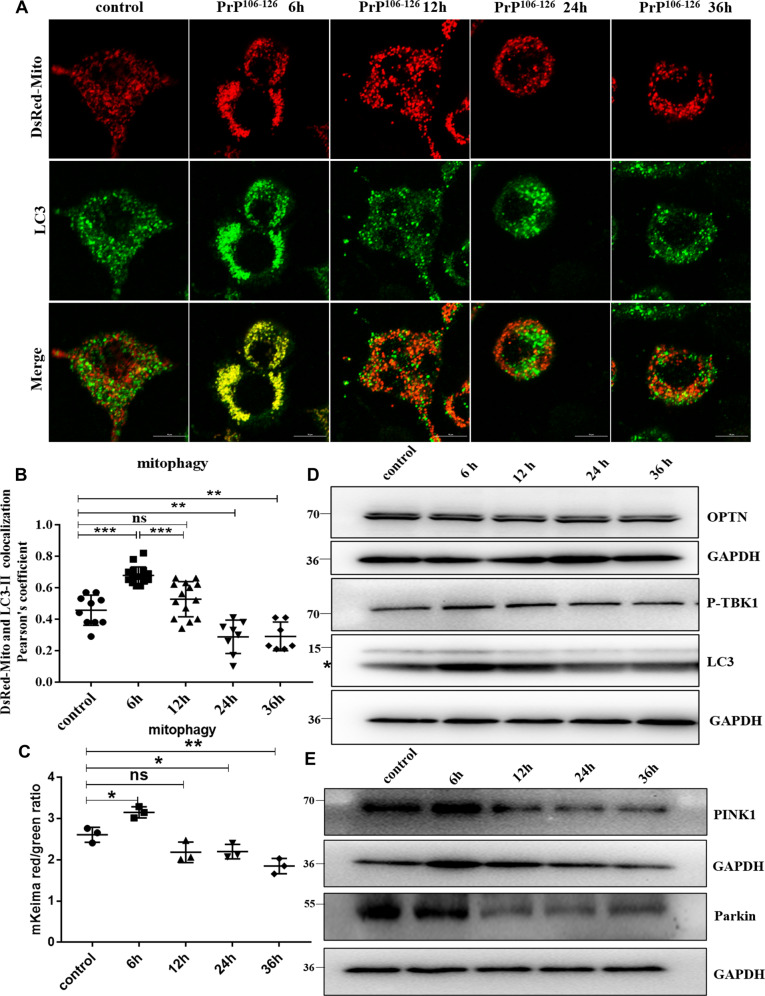
PrP106-126-induced mitophagy deficiency in N2a cells. **A** Mitophagy events through time show the colocalization between the autophagosome protein LC3-II and the mitochondria (labeled with DsRed-Mito) in N2a cells treated with PrP106-126. Scale bar: 10 µm. **B** Comparison of the localization of LC3-II and DsRed-Mito in cells in **A**. **C** Mitophagy in N2a cells treated with PrP106-126 was characterized by the COX8-mKeima fluorescence ratio change in Figure S1A. **D, E** Western blots of mitophagy-related proteins undergoing PrP106-126 treatments. GAPDH was used as the loading control. The “*“ in the Western blots band indicates LC3-II. Data were mean (SD); ns not significant; *P < 0.05; **P < 0.01; ***P < 0.001. All experiments were repeated at least three times.

Our previous results had shown that prion diseases are characterized by morphological mitochondrial fragmentation and dysfunction in the affected neurons [15, 28]. So here, we tested the changes in mitophagy with prolonged PrP106-126 treatment time to uncover the cellular and molecular causes of damaged mitochondrial accumulation in N2a cells. In order to detect mitophagy, the following two methods were used: (1) We took advantage of coral-derived protein Keima, within the acidic lysosome (pH 4.0) after mitophagy, mitochondrial matrix-targeted Keima (COX8-mKeima)-fluorescence would change from green to red [29]; (2) We also detected mitophagy by observation of colocalization changes between mitochondrial Marker DsRed-Mito and autophagosome protein LC3-II. Interestingly, as PrP106-126 incubation time progressed (6, 12, 24, and 36 h), the mitophagy level first showed a compensatory activation at 6 h, after which levels lowered significantly below normal (Fig. 1A–C and S1A). In addition, after 6 h of incubation with PrP106-126, P-TBK1 (TANK-binding kinase 1 phosphorylation at Ser172) (Fig. 1D and S1C) [30, 31], and LC3-II (microtubule-associated protein 1 light chain 3 beta) (Fig. 1D and S1D) proteins levels significantly increased, while OPTN (optineurin) had no significant difference (Fig. 1D and S1B) in the N2a cells. But these proteins showed a significant downward trend after treatment for 12 h, especially after 24 h treatment: P-TBK1 protein level decreased by about 43%, LC3-II protein level decreased by about 44%, and OPTN protein level decreased by about 38% (Fig. 1D and S1B–D). These results demonstrate that mitophagy in N2a cells was severely impaired after 12 h of PrP106-126 treatment.

### PINK1-parkin-related mitophagy defects in N2a cells are induced by PrP106-126

Parkin and PINK1 are two key molecules in mitophagy regulation [32, 33]. To further reveal the molecular cause of defective mitophagy, we examined F-PINK1 (full-length PINK1) and Parkin protein levels. F-PINK1 (Fig. 1E and S1E) levels increased slightly in N2a cells after 6 h of incubation with PrP106-126, but parkin showed no significant change (Fig. 1E and S1F). Consistently, both F-PINK1 and parkin levels were significantly reduced by about 44 and 41%, respectively, after PrP106-126 treatment for 12–36 h (Fig. 1E and S1E, F).

To further investigate whether mitophagy deficiency in prion diseases is due to PINK1-parkin pathway impairment, we chose two mitophagy activators, UA [12, 34] and NMN [12, 35, 36], that can activate the PINK1-parkin pathway. The UA (concentration ranges: 100, 50, 25 µM) and NMN (concentration ranges: 5, 2,5, 1 mM) treatments were not toxic to N2a cells (Figs. S2E, S3E). When N2a cells were incubated with PrP106-126 for 24 h, we found that added 2.5 mM NMN (Fig. S2A–D) and 50 µM UA (Fig. S3A–D) can alleviate the reduction of mitophagy-related protein levels induced by PrP106-126. Compared with the PrP106-126 treatment group, the protein levels of PINK1 and Parkin increased about 3-fold and LC3-II increased about 1.4-fold by adding 2.5 mM NMN; the addition of UA increased the PINK1 and Parkin protein levels by about 2.3-fold, and the LC3-II protein levels by about 1.9-fold. Altogether, our data suggest that neurotoxic peptides induce PINK1-parkin-mediated mitophagy defects in N2a cells, while NMN and UA can activate this mitophagy pathway in this disease model.

### PINK1 is required for parkin-mediated mitophagy in a prion disease model

**Fig. 2:**
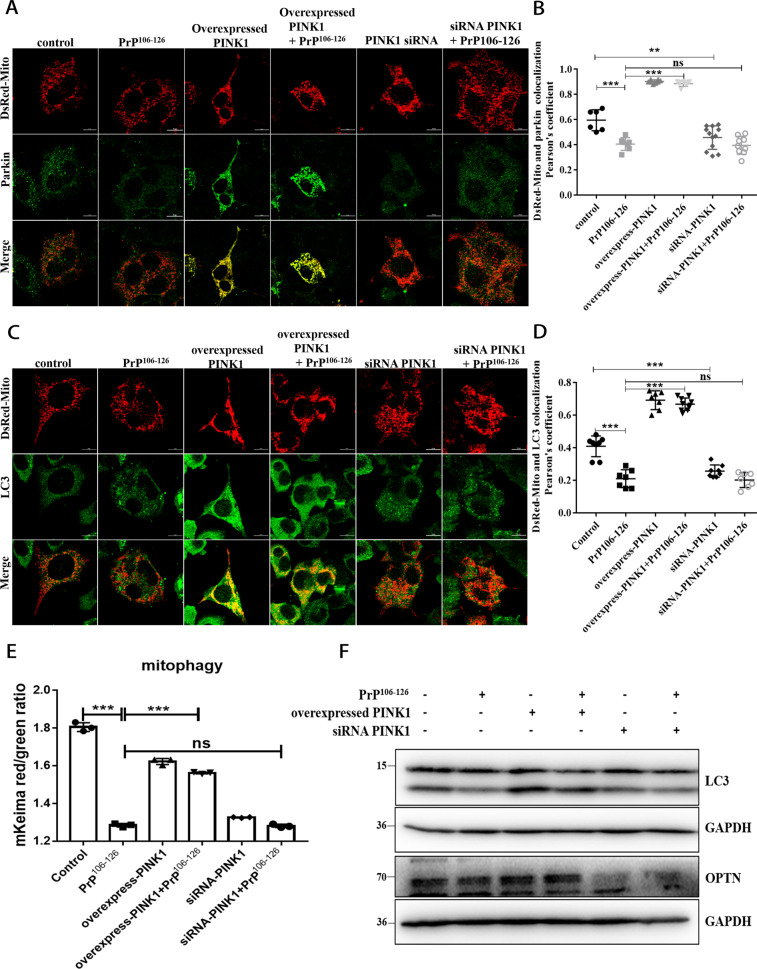
PINK1 is required for parkin-mediated mitophagy in a prion disease model. **A** Immunofluorescence images of mitochondrial Marker DsRed-Mito and parkin colocalization in N2a cells from Figure S4A–D with or without PrP106-126 treatment. Scale bar: 10 µm. **B** Comparisons of parkin recruitment in cells in **A**. **C** Immunofluorescence images of mitophagy events indicated by autophagosome protein LC3-II colocalization with mitochondria (labeled with DsRed-Mito) in N2a cells. Scale bar: 10 µm. **D** Comparison of the localization of LC3-II and DsRed-Mito in cells in **C**. **E** Mitophagy was characterized by the COX8-mKeima fluorescence ratio change in Fig. S4E. **F** Western blots of mitophagy-related proteins (LC3, OPTN) in overexpressed and knocked down (siRNA) F-PINK1 cells with or without PrP106-126 treatment. GAPDH was the loading control. Data were mean (SD). ns not significant; *P < 0.05; **P < 0.01; ***P < 0.001. All experiments were repeated at least three times.

Given the parkin/ PINK1 protein interaction in which parkin recruitment to mitochondria depends on PINK1 [19, 25, 26, 37], we sought to identify PINK1’s role in parkin recruitment and mitophagy in prion disease. So, we overexpressed (The level of PINK1 in PINK1-overexpressed cells was increased by about 36%) (Fig. S4A, B) or knocked down PINK1 (siRNA-PINK1, The level of PINK1 in PINK1- knockdown cells was reduced by about 66%) (Fig. S4C, D) in N2a cells and revealed that N2a cells treated with PrP106-126 for 24 h had significantly inhibited parkin recruitment to mitochondria, more parkin was recruited to the mitochondria in PINK1-overexpressed cells than to the mitochondria in PINK1 knockdown cells (Fig. 2A, B), thus suggesting that PINK1 overexpression alleviated the inhibition of parkin recruitment induced by PrP106-126, but PINK1 deficiency enhanced the inhibition. To further confirm the role of PINK1 in mitophagy, we examined COX8-mKeima dual fluorescence changes and the colocalization of mitochondrial and LC3-II, and found that cells with overexpressed PINK1 had mitochondria and LC3-II colocalization (Fig. 2C, D), and that mitophagy defect caused by PrP106-126 was alleviated (Fig. 2E and S4E). However, PINK1 knockdown inhibited the relocation of LC3-II to the mitochondria in N2a cells and the mitophagy defect was aggravated (Fig. 2C–E and S4E). Therefore, we used Western blotting to examine LC3-II (Fig. 2F and S5A) and OPTN (Fig. 2F and S5B) protein levels in those cells and found that overexpression PINK1 staunched PrP106-126-caused decreases (The LC3-II protein level increased by about 46%, and the OPTN protein level increased by about 23%). Not surprisingly, LC3-II and OPTN protein levels were significantly reduced by ~64 and 15% in PINK1 knockdown and PrP106-126 treated cells, respectively (Fig. 2F and S5A, B). The above results indicate that PINK1 is required for Parkin to recruit to mitochondria and mediate mitophagy in prion disease.

### Mitophagy defects caused by PrP106-126 can be alleviated by NMN, UA, and overexpression of Parkin

**Fig. 3:**
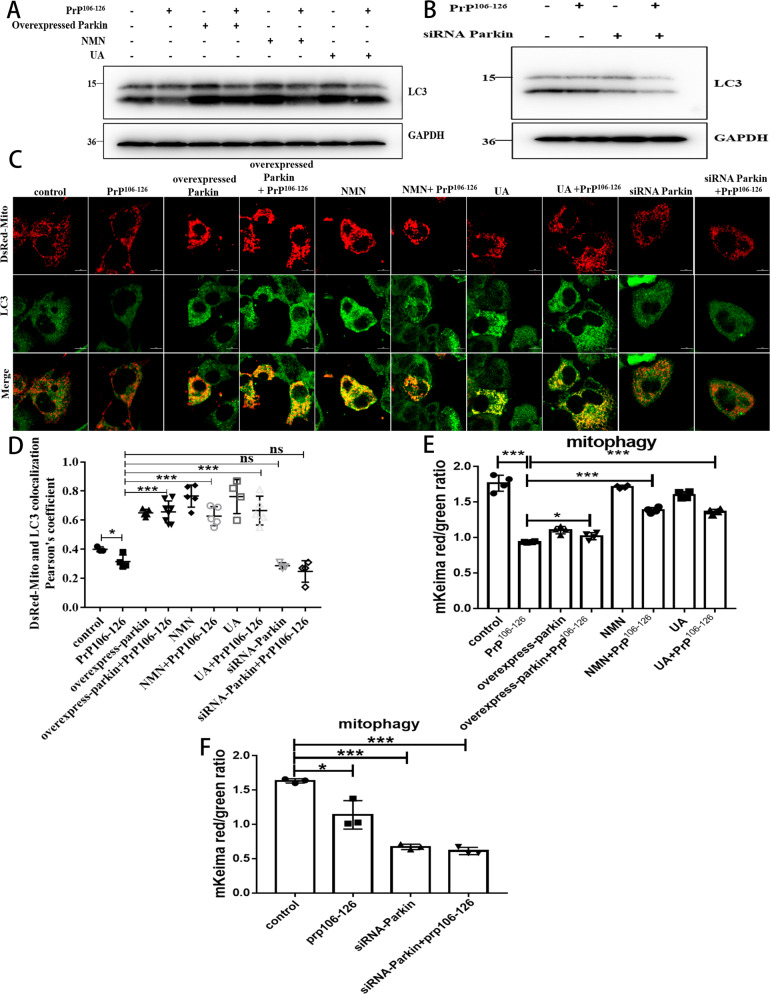
Mitophagy defects caused by PrP106-126 can be alleviated by NMN, UA, and overexpression of Parkin. **A**, **B** Western blots of LC3-I and LC3-II levels in N2a cells from Figure S6A–D, with and without PrP106-126, nicotinamide mononucleotide (NMN), and urolithin A (UA) treatments. **C** Immunofluorescence images of mitophagy events indicated by the colocalization of autophagosome protein LC3-II and the mitochondria (labeled with DsRed-Mito). Scale bar: 10 µm. **D** Comparison of the localization of LC3-II and DsRed-Mito in cells from **C**. **E**, **F** Mitophagy was characterized by the COX8-mKeima fluorescence ratio change in Figure S7A, B. Data were mean (SD). ns not significant; *P < 0.05; **P < 0.01; ***P < 0.001. All experiments were repeated at least three times.

Next, we overexpressed (Fig. S6A, B, The level of Parkin in Parkin-overexpressed cells was increased by about 81%) and suppressed (Fig. S6C, D, The level of Parkin in Parkin knockdown cells was reduced by about 52%) the PINK1 downstream effector molecule parkin, an important mitophagy signal amplifier in the PINK1-parkin pathway [19], and used mitophagy inducers UA (50 µM) and NMN (2.5 mM) to verify their effects on mitophagy in prion disease. To do that, we quantified autophagic flux on Western blots, compared with the control group, the LC3-II/LC3-I of the PrP106-126-treated neurons was significantly reduced by about 35% (Fig. 3A, B and S6E, F), and the decrease in autophagy flux was aggravated in parkin knockdown cells, which was reduced by about 72% (Fig. 3B and S6F), LC3-II/LC3-I reduction was blocked in overexpressed parkin, and in NMN- and UA-supplemented cells, compared with the PrP106-126 treatment group, the LC3-II/LC3-I protein levels increased by about 50, 35, and 45%, respectively (Fig. 3A and S6E). Furthermore, both overexpressing parkin or using either UA or NMN can promote mitochondria and LC3-II colocalization (Fig. 3C, D) and can alleviate mitophagy defects caused by PrP106-126 (Fig. 3E and S7A). Conversely, deleting parkin in PrP106-126-treated N2a cells blocked the relocation of LC3-II to the mitochondria (Fig. 3C, D) and potently suppressed mitophagy (Fig. 3F and S7B). Thus, the overexpression of Parkin and the use of mitophagy activator NMN, UA can alleviate the mitophagy defect caused by PrP106-126.

### NMN supplementation and Parkin overexpression can reduce the accumulation of damaged neuronal mitochondria caused by PrP106-126

**Fig. 4:**
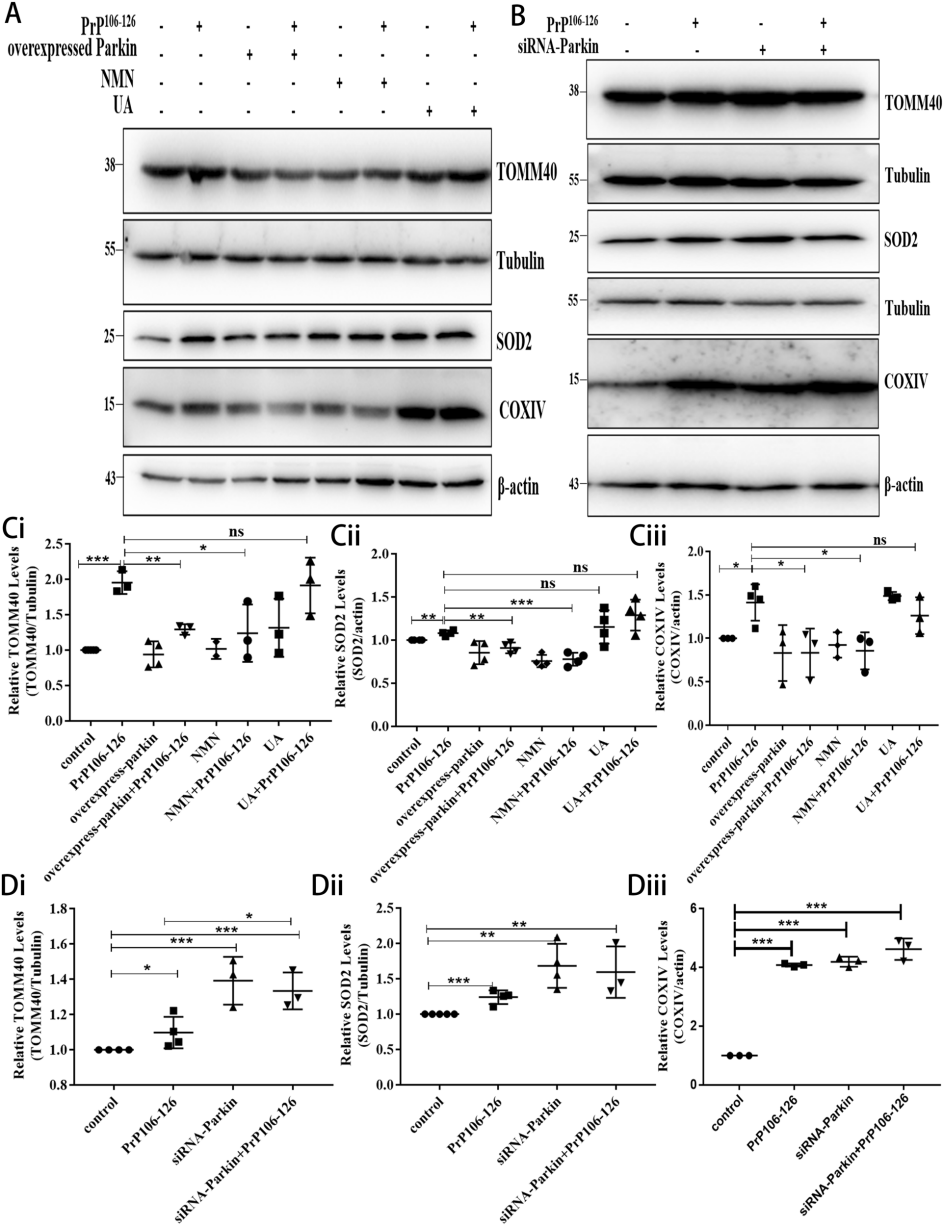
NMN supplementation and Parkin overexpression can reduce the accumulation of damaged neuronal mitochondria caused by PrP106-126. **A**, **B** Western blots of mitochondrial protein (TOMM40, COXIV, SOD2) expressions in N2a cells with **A** overexpressed and **B** knocked down (siRNA) parkin cells, with and without PrP106-126, nicotinamide mononucleotide (NMN), and urolithin A (UA) treatments (Tubulin and β-actin served as loading controls.) **C**, **D** Comparisons of mitochondrial protein levels, relative to controls, in cells from **A** and **B**, respectively. Data were mean (SD). ns not significant; *P < 0.05; **P < 0.01; ***P < 0.001. All experiments were repeated at least three times.

Mitophagy defects lead to an accumulation of damaged mitochondria [18, 38] and we have demonstrated PINK1-parkin-mediated mitophagy impairment in a prion disease model. Therefore, we next examined whether neuronal mitophagy activated through pharmacological or genetic interventions would affect the clearance of damaged mitochondria. So, the degradation of mitochondrial proteins TOMM40 (outer mitochondrial membrane protein), COXIV (inner mitochondrial membrane protein), and SOD2 (mitochondrial matrix protein) was analyzed by Western blotting. Compared to mitochondrial protein levels in the control group, those levels in N2a cells incubated with PrP106-126 for 24 h were significantly greater (Fig. 4A–D), the TOMM40 protein level increased by about 1.9-fold, the SOD2 protein level increased by about 1.2-fold, and the COXIV protein level increased by about 1.4-fold, thus indicating that PrP106-126 treatment significantly inhibited the elimination of damaged mitochondria. Mitochondrial protein levels decreased significantly after PrP106-126 treatment in parkin-overexpressed (The protein level of TOMM40 was reduced by ~66%, the protein level of SOD2 was reduced by ~17%, and the protein level of COXIV was reduced by ~58%) and NMN-supplemented cells (The protein level of TOMM40 was reduced by ~71%, the protein level of SOD2 was reduced by ~30%, and the protein level of COXIV was reduced by ~56%) (Fig. 4A, C), but not in parkin knockdown cells (Fig. 4B, D). Intriguing, after using UA, TOMM40, SOD2, and COXIV proteins increased significantly in neurons compared with the control group, UA supplementation did not significantly alleviate the accumulation of damaged mitochondria induced by PrP106-126 (Fig. 4A, C). These results suggest that parkin overexpression and use of NMN can activate mitophagy and significantly reduce the accumulation of damaged mitochondria induced by PrP106-126. Although UA addition can activate mitophagy, it does not significantly alleviate mitochondrial accumulation in a prion disease model.

### NMN supplementation and Parkin overexpression can alleviate PrP106-126-induced morphological mitochondrial damage and dysfunction

**Fig. 5:**
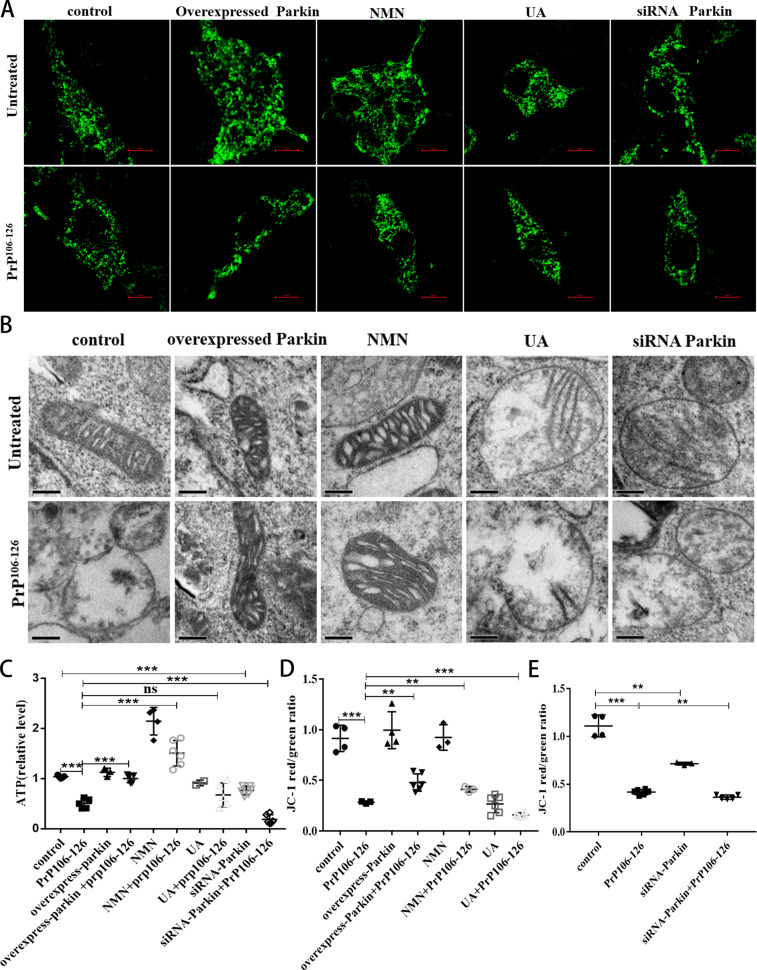
NMN supplementation and Parkin overexpression can alleviate PrP106-126-induced morphological mitochondrial damage and dysfunction. **A** Immunofluorescence images of Mito-GPF-tagged mitochondria showing differing morphologies after various treatments. Scale bar: 10 µm. **B** Mitochondrial ultrastructure of cells observed using transmission electron microscopy. Scale bar: 200 nm. **C** Comparisons of ATP levels in N2a cells. **D**, **E** Comparisons of the analyzed MMP data, as measured by changes in red/green intensity ratios, in cells in Figure S8C, D. Data were mean (SD). ns not significant; *P < 0.05; **P < 0.01; ***P < 0.001. All experiments were repeated at least three times.

Since in vivo and in vitro models of prion disease, mitochondrial fragmentation, cristae loss, and ATP depletion have been found [15, 28, 39], we wanted to verify the effect of parkin overexpression and of the use of mitophagy activators UA and NMN on mitochondrial morphology and function. We found that PrP106-126 induced severe mitochondrial fragmentation in N2a cells (Fig. 5A and S8A). Cells with overexpressed parkin or with added NMN had longer neuronal mitochondria, but parkin deficiency destabilized the mitochondrial network in neurotoxic peptide-treated cells (Fig. 5A and S8A). Next, using electron microscopy, we found abnormal mitochondrial ultrastructure (reduced cristae and swelling) in N2a cells treated with neurotoxic peptides, but both overexpressed parkin and supplemented NMN remodeled mitochondrial cristae and reduce swelling (Fig. 5B and S8B). We also detected fragmented mitochondrial cristae in both parkin-deficient and PrP106-126-treated cells (Fig. 5B and S8B). Consistent with the above results (Fig. 4A, C), UA did not relieve mitochondrial fragmentation (Fig. 5A and S8A), swelling, and cristae loss (Fig. 5B and S8B) caused by PrP106-126.

Next, we examined how restoring defective mitochondrial morphology affects mitochondrial function. Indeed, in N2a cells with mitophagy defects from PrP106-126 treatment, we noted decreased intracellular ATP levels (Fig. 5C) and mitochondrial membrane depolarization (MMP) (Fig. 5D, E and S8C–D) that became exacerbated in parkin knockdown cells (Fig. 5C, E and S8D). However, parkin overexpression and NMN supplementation prevented both prion-induced ATP loss (Fig. 5C) and reduced MMP (Fig. 5D and S8C). Consistent with previous studies [34], UA alone reduced ATP levels and MMP but also aggravated mitochondrial dysfunction induced by PrP106-126 (Fig. 5C, D and S8C). These findings, combined with the distorted mitochondrial network morphology noted above, indicate that mitophagy impairment precipitates pronounced mitochondrial dysfunction in prion disease. While parkin overexpression and NMN supplementation can alleviate mitochondrial morphological damage and dysfunction, the UA mitophagy activation mechanism may aggravate the mitochondrial network morphology fragmentation and dysfunction caused by PrP106-126.

### Activation of PINK1-parkin-mediated mitophagy attenuates PrP106-126-induced neuronal apoptosis

**Fig. 6:**
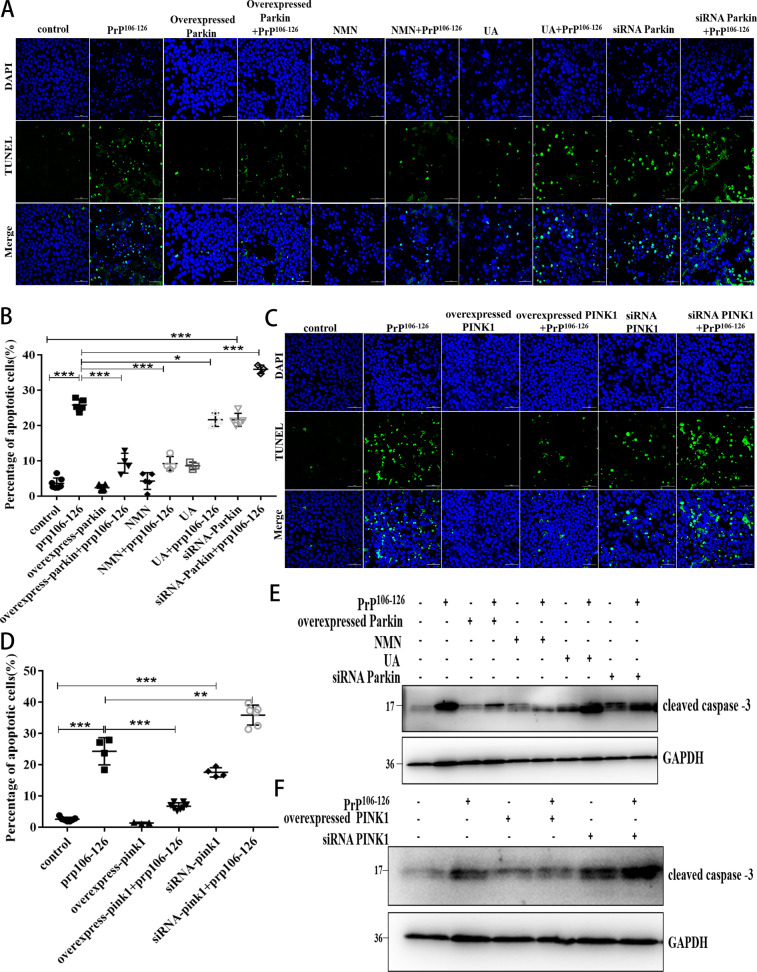
Activation of PINK1-parkin-mediated mitophagy attenuates PrP106-126-induced neuronal apoptosis. **A**, **C** Fluorescence images of cell apoptosis detected by TUNEL assays (green stain) in control or treated N2a cells. DAPI (blue) indicates intact cells. Scale bar: 50 µm. **B**, **D** Comparisons of the proportions of apoptotic cells to all cells in each group in **A** and **C**, respectively. **E**, **F** Western blots of the cleaved caspase 3 levels in treated cells. GAPDH was used as the loading control. Data were mean (SD). ns not significant; *P < 0.05; **P < 0.01; ***P < 0.001. All experiments were repeated at least three times.

So far, our findings suggest that pharmacological and genetic interventions that activate mitophagy can reduce the neurotoxic peptide-induced persistent accumulation of damaged mitochondria. However, we had to investigate the possible effects that both overexpressed PINK1 and parkin and the use of mitophagy activators UA and NMN might have on neuronal cells survival. We found that N2a cells with overexpressed parkin or PINK1, and added NMN all experienced inhibited PrP106-126-induced apoptosis (Fig. 6A–D). In contrast, parkin and PINK1 knockdown significantly increased PrP106-126-induced apoptosis, and UA could not significantly alleviate PrP106-126-induced apoptosis (Fig. 6A–D). Since damaged mitochondria can activate caspase 3–dependent neuronal apoptosis by releasing cytochrome c, we tested caspase 3 activation and found that parkin or PINK1 overexpression, as well as the use of NMN, significantly reduced PrP106-126-induced caspase 3 activation, and the protein levels of cleaved caspase 3 were reduced by about 50, 23, and 29%, respectively. but that effect did not occur in knockdown cells (Fig. 6E, F and S9A, B). UA didn’t alleviate PrP106-126-induced neuronal apoptosis (Fig. 6E and Fig. S9A). Finally, we analyzed changes in neuronal cells survival and found that the percentage of surviving neurons, relative to PrP106-126-treated neurons, increased significantly with overexpressed parkin and PINK1 and with NMN supplementation, but not in knockdown cells (Fig. S9C, D). UA alone had no effect on neuronal activity, and neither did it alleviate the decreased neuronal activity caused by PrP106-126 (Figure S9C). Altogether, mitophagy activation can reduce neuronal apoptosis caused by PrP106-126.

## Discussion

Since mitochondrial damage is an early indicator of neuronal damage, discovering the connections between mitophagy deficiency and mitochondrial damage is very important for the early prevention and treatment of prion diseases. In this study, with PrP106-126 treatment, N2a cells underwent significant apoptosis, cell viability decreased to 50–60%, and mitochondria were obviously fragmented, at the same time, we found that PINK1-Parkin-mediated mitophagy was also significantly defective. Additionally, defects in this conserved mitophagy pathway have been reported in other diseases, (e.g., AD, PD, and age-related cardiomyopathy) [12, 16, 18, 40–43]. This suggests that in the prion disease model, the accumulation of damaged mitochondria may be related to the mitochondrial quality control pathway-mitophagy defect.

To further verify our findings, we used pharmacology and gene intervention to regulate the expression of PINK1, Parkin, the key factors of mitophagy. We found that overexpressed PINK1 can alleviate inhibited Parkin recruitment and impaired mitophagy caused by PrP106-126, but not by PINK1 deficiency. Thus, PINK1 is required for parkin to recruit to the mitochondria and mediate mitophagy in a prion disease model.

Similarly, the overexpression of Parkin and the supplementation of mitophagy activator NMN can also alleviate mitophagy deficiency, mitochondrial morphological damage and dysfunction, and apoptosis caused by PrP106-126, while knocking down parkin exacerbates those injuries. A study has reported that in prion-infected cells and experimental mouse models, despite a large accumulation of damaged mitochondria and autophagosomes and severe neuronal damage, PINK1-parkin-mediated mitophagy still seems to show a protective role in deleterious situations [44]. Combined with our research, pharmacological and genetic overexpression of PINK1 and Parkin can significantly alleviate neuronal apoptosis and death in prion disease, suggesting that mitochondrial dysfunction and bioenergy deficiency in prion disease may be alleviated by stimulating PINK1-parkin-mediated mitophagy, thereby fostering a healthy mitochondrial pool and ultimately significantly increasing neuronal activity. Additionally, studies of other neurodegenerative disease models confirmed that *PINK1, parkin* gene interventions also affect pathological changes caused by those diseases [27, 40, 45, 46]. At present, many studies have reported molecular components such as the adenine nucleotide translocator (ANT) complex regulating PINK1 stability in MOM and recruiting parkin [47–49]. Obviously then, further studies need to explore the molecular mechanisms regulating PINK1-parkin-mediated mitophagy in prion diseases.

However, we found an interesting phenomenon that mitophagy activator UA, cannot alleviate mitochondrial fragmentation and dysfunction caused by PrP106-126, which eventually leads to cell apoptosis and decreased vitality. Based on reported studies [34], UA’s ability to prolong life and alleviate aging depends on mitochondrial function. We speculate that cells affected by prion disease have severe mitochondrial morphological fragmentation and dysfunction, so although UA can activate mitophagy, it also enhances damaged mitochondrial fragmentation. Then in that case, few functional mitochondria are left for UA to function in the neurons affected by the disease. Therefore, UA supplementation will not alleviate neuronal apoptosis and death caused by prion disease. Another mitophagy activator, NMN supplementation could not only activate PINK1-parkin-mediated mitophagy in the prion disease cell model but also restore mitochondrial morphology and function, thereby alleviating neuronal apoptosis induced by PrP106-126, the therapeutic effect of NMN has also been reported in other diseases, such as premature aging-related ataxia telangiectasia and hypertension-related stroke [50, 51]. The different mechanisms of NMN and UA in activating mitophagy require us to further study, which may help us find precise targets for the treatment of prion diseases.

In summary, our results showed that pharmacological and genetic interventions can not only alleviate defected mitophagy caused by prion diseases but can also protect neurons from apoptosis. Further therapeutic approaches aimed at modulating PINK1-parkin-mediated mitophagy may help attenuate the mitochondrial pathology associated with prion disease.

## Materials and methods

### Cell culture and treatment

N2a cells were purchased from Cell Resource Center (IBMS, CAMS/PUMC, China), and tested to be mycoplasma free. N2a cells were cultured in Gibco DMEM (catalog no. C11995500BT) supplemented with 10% (v/v) fetal bovine serum (Gibco, NY, USA) at 37 °C with 5% CO_2_ in a humid incubator.

We obtained synthesized PrP106-126 peptide (KTNMKHMAGAAAAGAVVGGLG; > 98% purity) from YaMei Peptides Bio-Tech. It was dissolved in PBS to a 1 mM concentration and shaken to aggregate at 4 °C for 24 h. Experiments were conducted with a final peptide concentration of 100 μM.

### Western blotting

Western blotting was performed following previous laboratory procedures [15, 28, 39]. The following primary antibodies were used for blotting: anti-parkin antibody (Abcam, catalog no. ab77924; Santa Cruz Biotechnology, catalog no. sc-32282), PINK1 antibody (Novus Biologicals, catalog no. BC100–494), caspase 3 polyclonal antibody (Proteintech, catalog no. 19677-1-AP), anti-optineurin antibody [EPR20654] (Abcam, catalog no. ab213556), phospho-TBK1/NAK (Ser172) (D52C2) XP rabbit monoclonal antibody (Cell Signaling Technology, catalog no. 5483), LC3 rabbit polyclonal antibody (Proteintech, catalog no. 14600-1-AP), TOMM40 rabbit polyclonal antibody (Proteintech, catalog no. 18409-1-AP), SOD2 rabbit polyclonal antibody (Proteintech, catalog no. 24127-1-AP), COXIV rabbit polyclonal antibody (Proteintech, catalog no. 11242-1-AP), GAPDH monoclonal antibody (Proteintech, catalog no. 60004-1-Ig), beta-actin monoclonal antibody (Proteintech, catalog no. 66009-1-Ig), and alpha-tubulin polyclonal antibody (Proteintech, catalog no. 11224-1-AP). Secondary antibodies included HP-goat anti-mouse (ZsBio, catalog no. ZB-2305Beijing, China) and HP-goat anti-rabbit (ZsBio, catalog no. ZB-2301). The blots were visualized using a 5200 Chemiluminescent Imaging System (Tanon Science and Technology).

### Plasmids and transfection

PINK1 siRNA (sense:5′-CUAUGAAAUCUUUGGGCUUTT-3′; antisense: 5′-AAGCCCAAAGAUUUCAUAGTT-3′), parkin siRNA (sense: 5′-GAGUGGUGAGUGCCAGUCUTT-3′; antisense: 5′-AGACUGGCACUCACCACUCTT-3′), plasmid pcDNA3.1(+)-PINK1 and -parkin were obtained from Synbio Technologies. The Mito-GFP and DsRed-Mito plasmids were obtained from Clontech. The pSLenti-CMV-mt-mKeima-PGK-Puro-WPRE were obtained from Obio Technology. N2a cells were transfected using Lipofectamine 3000 (Invitrogen, catalog no. L3000015) in Opti-MEM (Gibco, catalog no. 31985062) following the manufacturers’ instructions.

### Immunofluorescence microscopy

#### For fluorescence images of mitochondria

N2a cells were transfected with Mito-GFP for 48 h before being treated. Fluorescence images of mitochondria were acquired using an A1 confocal microscope (Nikon, Tokyo) and mitochondrial lengths were measured using ImageJ.

#### For mitophagy or parkin recruitment

N2a cells were transfected with DsRed-Mito, Plasmid DNA, or siRNA before being treated. The cells were then fixed, permeabilized, and sealed. The primary antibody was incubated overnight at 4 °C, and the next day the cells were incubated with fluorescent secondary antibodies Alexa Fluor 488 labeled goat anti-rabbit IgG (H + L) (Beyotime, catalog no. A0423) and Alexa Fluor 488 labeled goat anti-mouse IgG (H + L) (Beyotime, catalog no. A0428) for 1 h in the dark at 37 °C. Fluorescence images were visualized using a Nikon A1 confocal microscope.

### MMP detection

MMP was measured using an MMP assay kit with JC-1 (Beyotime, catalog no. C2006) according to the manufacturer’s instructions. Fluorescence signals were analyzed by a FACS Calibur (BD Biosciences, San Jose, CA, USA).

### ATP level detection

ATP levels were evaluated using an ATP Assay Kit (Beyotime, catalog no. S0027) according to the manufacturer’s instructions. Luminescence was measured using a GloMax 96 Microplate Luminometer (Promega, Madison, WI, USA).

### TUNEL assay

The One Step TUNEL Apoptosis Assay Kit (Beyotime, catalog no. C1086) was used according to the manufacturer’s instructions to detect N2a cell apoptosis. The cells were visualized using an A1 confocal microscope (Nikon).

### Cell viability assay

Neuron cell viability was evaluated using the Cell Counting Kit-8 assay (Beyotime, catalog no. C0038) according to the manufacturer’s instructions. A microplate reader (Thermo Fisher, Waltham, MA, USA) was used to obtain the samples’ absorbances at 450 nm, and cell viability was expressed as the percent of the untreated control.

### Statistical analyses

All assays were repeated at least three times, the number of replicates was presented by individual data points in each graph, and the data were expressed as mean (SD). Parametric data were analyzed using a two-tailed unpaired Student’s *t*-test and with a significance threshold of *P* < 0.05. Analysis of variance followed by *F*-test. Statistical analyses were performed using either Prism software version 7.0 (GraphPad, La Jolla, CA, USA) or ImageJ (National Institutes of Health, USA).

## Supplementary information

Supplementary figures

Original Data File

Original Data File

Original Data File

payment information

third-party declarations

Reproducibility checklist

## Data Availability

The data that supports the findings of this study are available in this published article and its supplementary information files include the following: Supplementary figures, Original date file-Western blot, Original date file-Flow cytometry, Original date file-Fluorescence confocal and Electron microscope.
