# Supplementary material for: PINK1-parkin-mediated neuronal mitophagy deficiency in prion disease
Source: Cell Death Dis. 2022 Feb 18;13(2):162. doi: 10.1038/s41419-022-04613-2 (PMC8858315; doi:10.1038/s41419-022-04613-2)
Supplement: Supplementary file 1 — Supplementary figures [file 41419_2022_4613_MOESM1_ESM.docx]

**Figure S1**


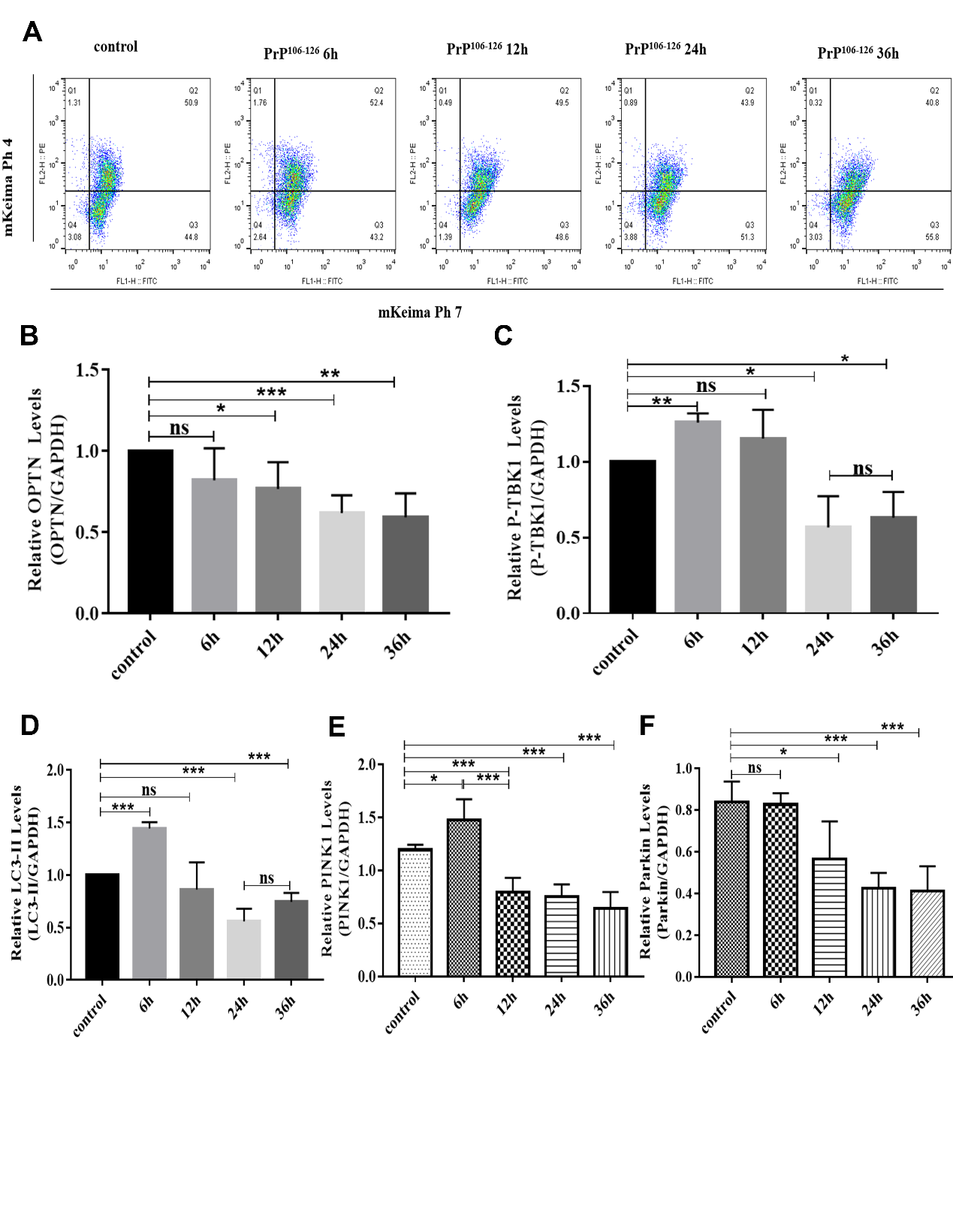


Figure S1. PINK1-parkin mediated mitophagy defects induced by PrP106-126 in N2a cells. (**A**) Mitophagy in N2a cells treated with PrP106-126 was characterized by the COX8-mKeima fluorescence ratio change. When mitophagy was activated, the fluorescence intensity of COX8- mKeima shifted from green (PH 7) to red (PH 4). (**B-F**) Comparisons of mitophagy-related protein levels, relative to control levels (GAPDH), in N2a cells in **Figure 1 D, E** Data are mean (SD). ns, not significant; **P* < 0.05; ***P* < 0.01; ****P* < 0.001. All experiments were repeated at least three times.

**Figure S2**


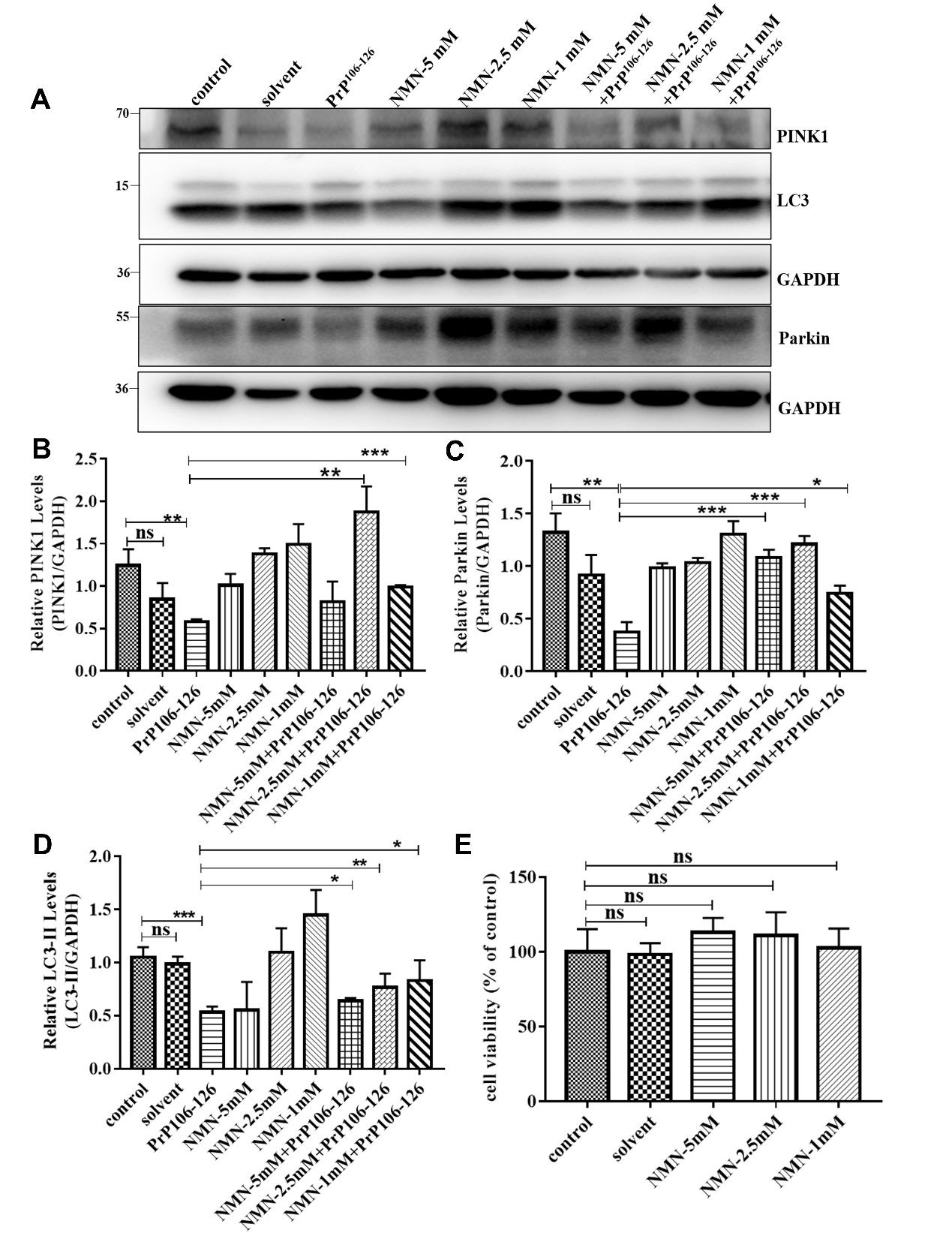


Figure S2. Mitophagy inducer NMN can activate PINK1-Parkin-mediated mitophagy. (**A**) Western blots of mitophagy-related proteins after PrP106-126, and nicotinamide mononucleotide (NMN) treatments. (**B-D**) Comparisons of the levels of mitophagy-related proteins, relative to control levels, in N2a cells from **A**. (**E**) Comparisons of cell activities of NMN-treated cells were detected using Cell Counting Kit-8 (CCK-8) assays. Cell viabilities were expressed as percentages of the untreated controls. Data are mean (SD). ns, not significant; **P* < 0.05; ***P* < 0.01; ****P* < 0.001. All experiments were repeated at least three times.

**Figure S3**


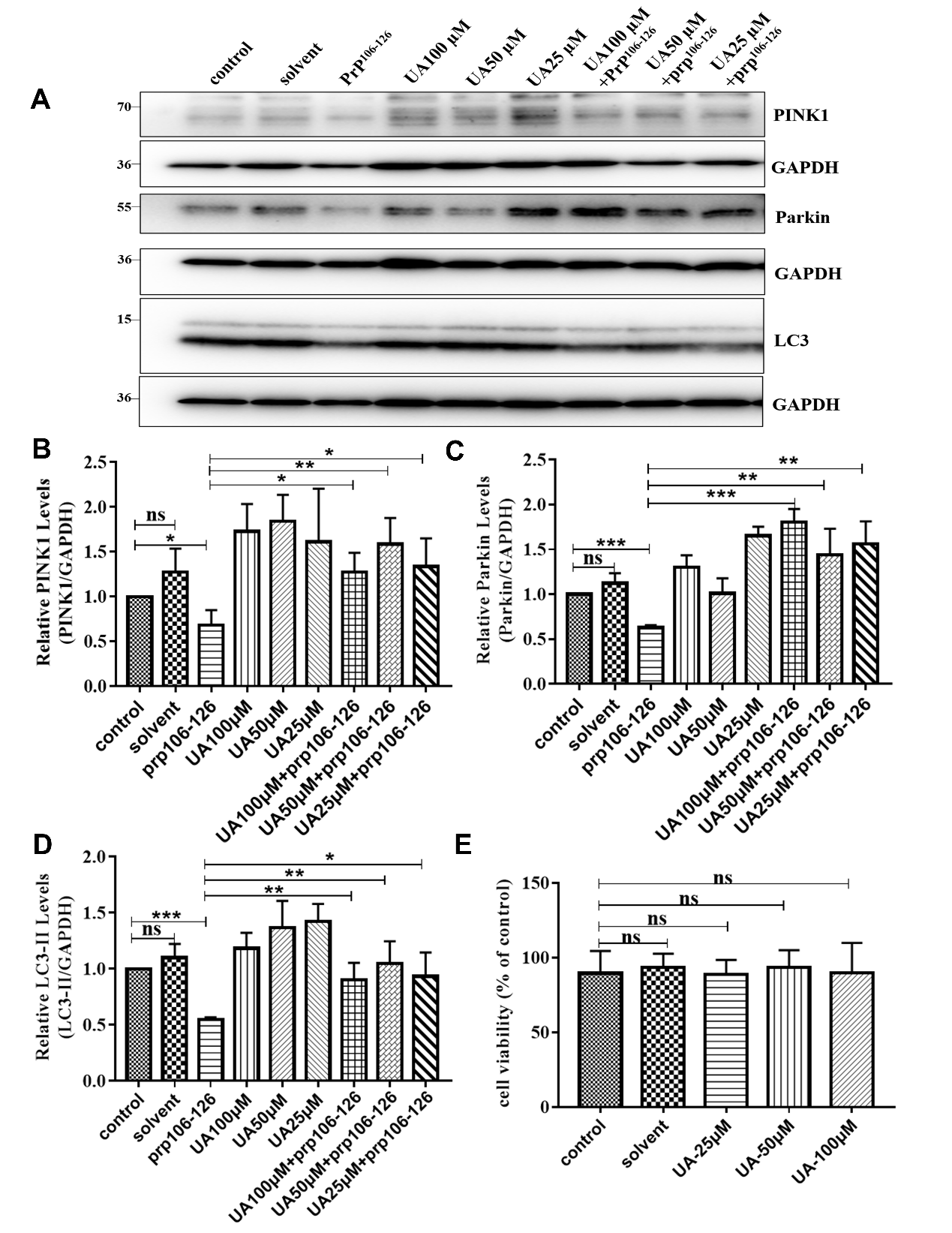


Figure S3. Mitophagy inducer UA can activate PINK1-Parkin-mediated mitophagy. (**A**) Western blots of mitophagy-related proteins after PrP106-126, and urolithin A (UA) treatments. (**B-D**) Comparisons of the levels of mitophagy-related proteins, relative to control levels, in N2a cells from **A**. (**E**) Comparisons of cell activities of UA-treated cells were detected using Cell Counting Kit-8 (CCK-8) assays. Cell viabilities were expressed as percentages of the untreated controls. Data are mean (SD). ns, not significant; **P* < 0.05; ***P* < 0.01; ****P* < 0.001. All experiments were repeated at least three times.

**Figure S4**


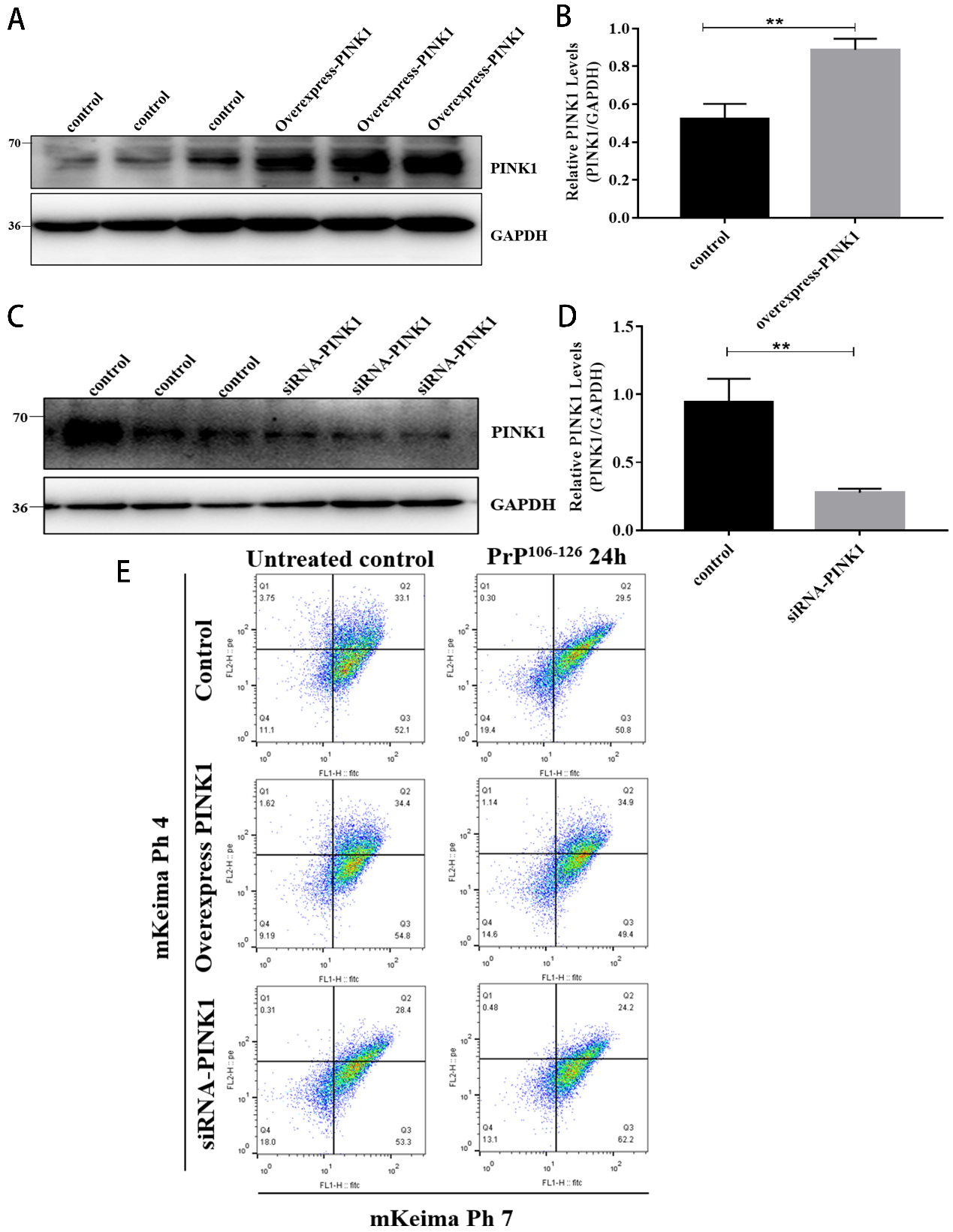


Figure S4. PINK1 is required for parkin-mediated mitophagy in prion disease model. **(A, C)** Western blots of overexpressed and knocked down (siRNA) F-PINK1 protein expression. GAPDH was the loading control. **(B, D)** Comparisons of F-PINK1 protein levels, relative to controls, in **A, C**. **(E)** Mitophagy was characterized by the COX8-mKeima fluorescence ratio change in N2a cells from **Figure S4A-D** with or without PrP106-126 treatment. Data are mean (SD). ns, not significant; **P* < 0.05; ***P* < 0.01; ****P* < 0.001. All experiments were repeated at least three times.

**Figure S5**


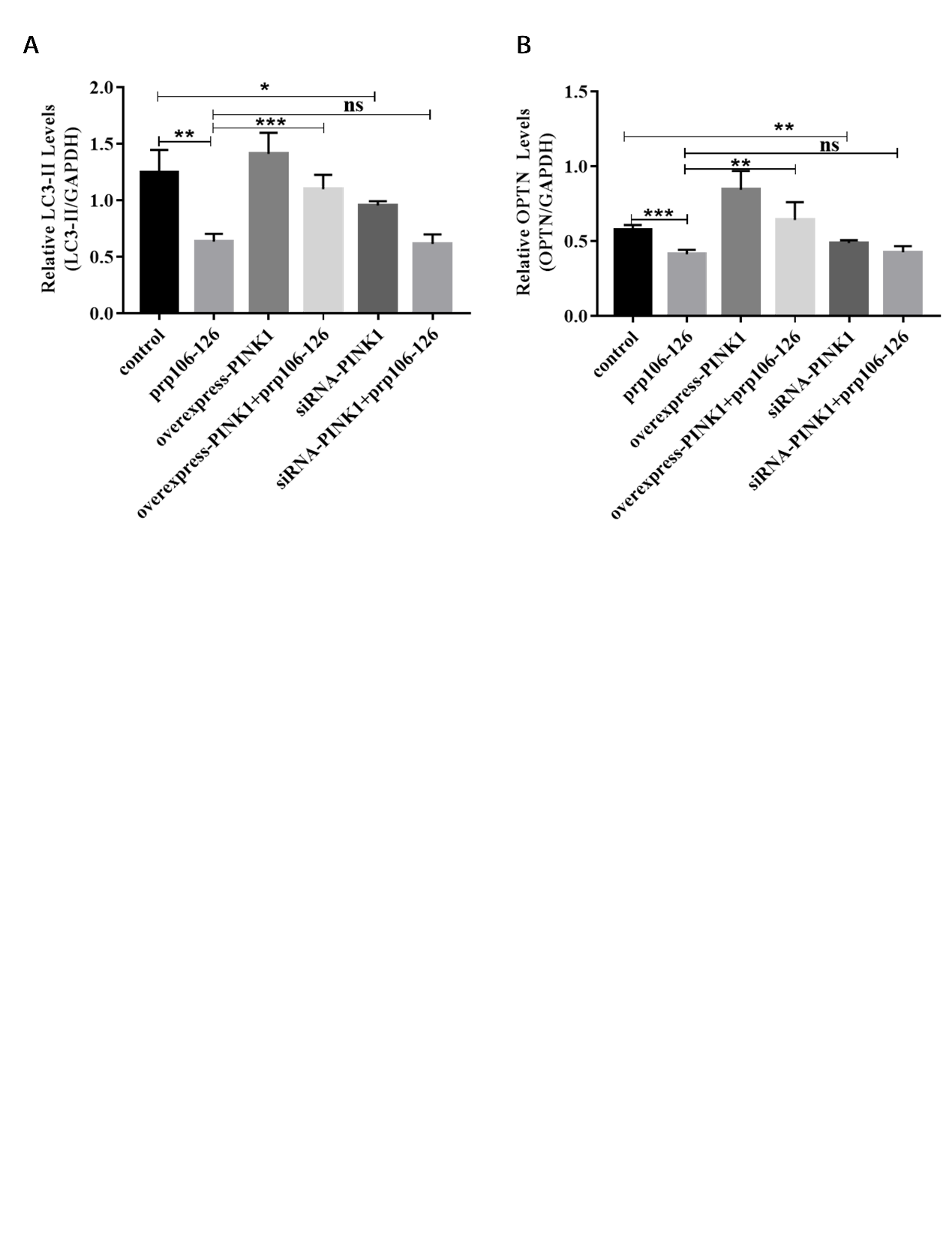


Figure S5. PINK1 is required for parkin-mediated mitophagy in prion disease model. **(A, B)** Comparisons of mitophagy-related protein levels, relative to controls, in cells from **Figure 2F**. Data are mean (SD). ns, not significant; *P < 0.05; **P < 0.01; ***P < 0.001. All experiments were repeated at least three times.

**Figure S6**


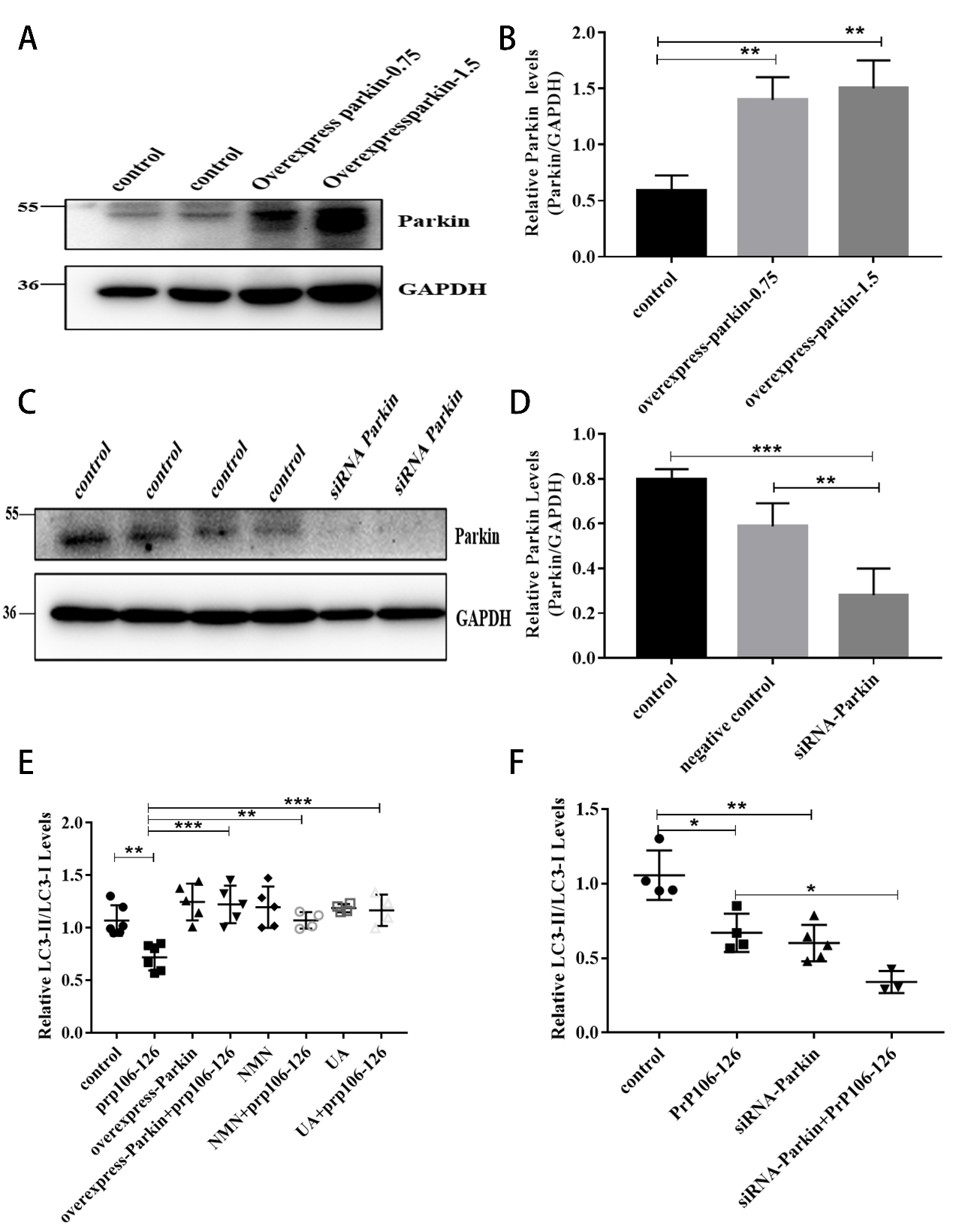


Figure S6. Mitophagy defects caused by PrP106-126 can be alleviated by NMN, UA and overexpression of Parkin. **(A, C)** Western blots of overexpressed and knocked down (siRNA) parkin protein. GAPDH was used as the loading control. **(B, D)** Comparisons of parkin protein levels, relative to controls, in cells from **A, C**. **(E, F)** Comparisons of LC3-II/LC3-I levels protein levels, relative to controls, in cells from **Figure 3A, B.** Data are mean (SD). ns, not significant; **P* < 0.05; ***P* < 0.01; ****P* < 0.001. All experiments were repeated at least three times.

**Figure S7**


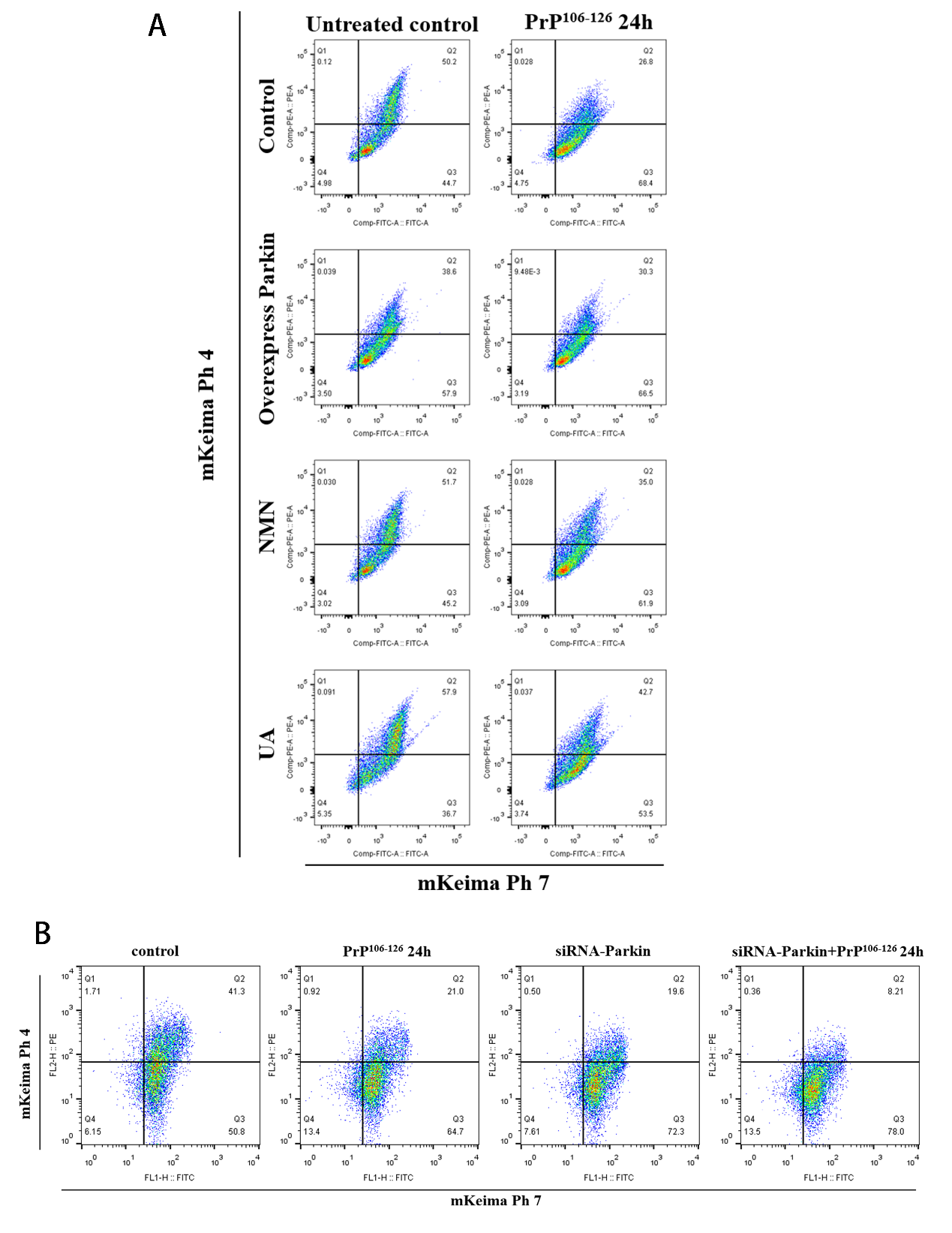


Figure S7. Mitophagy defects caused by PrP106-126 can be alleviated by NMN, UA and overexpression of Parkin. **(A, B)** Mitophagy was characterized by the COX8-mKeima fluorescence ratio change in N2a cells from **Figure S6A-D**, with and without PrP106-126, nicotinamide mononucleotide (NMN), and urolithin A (UA) treatments. All experiments were repeated at least three times.

**Figure S8**


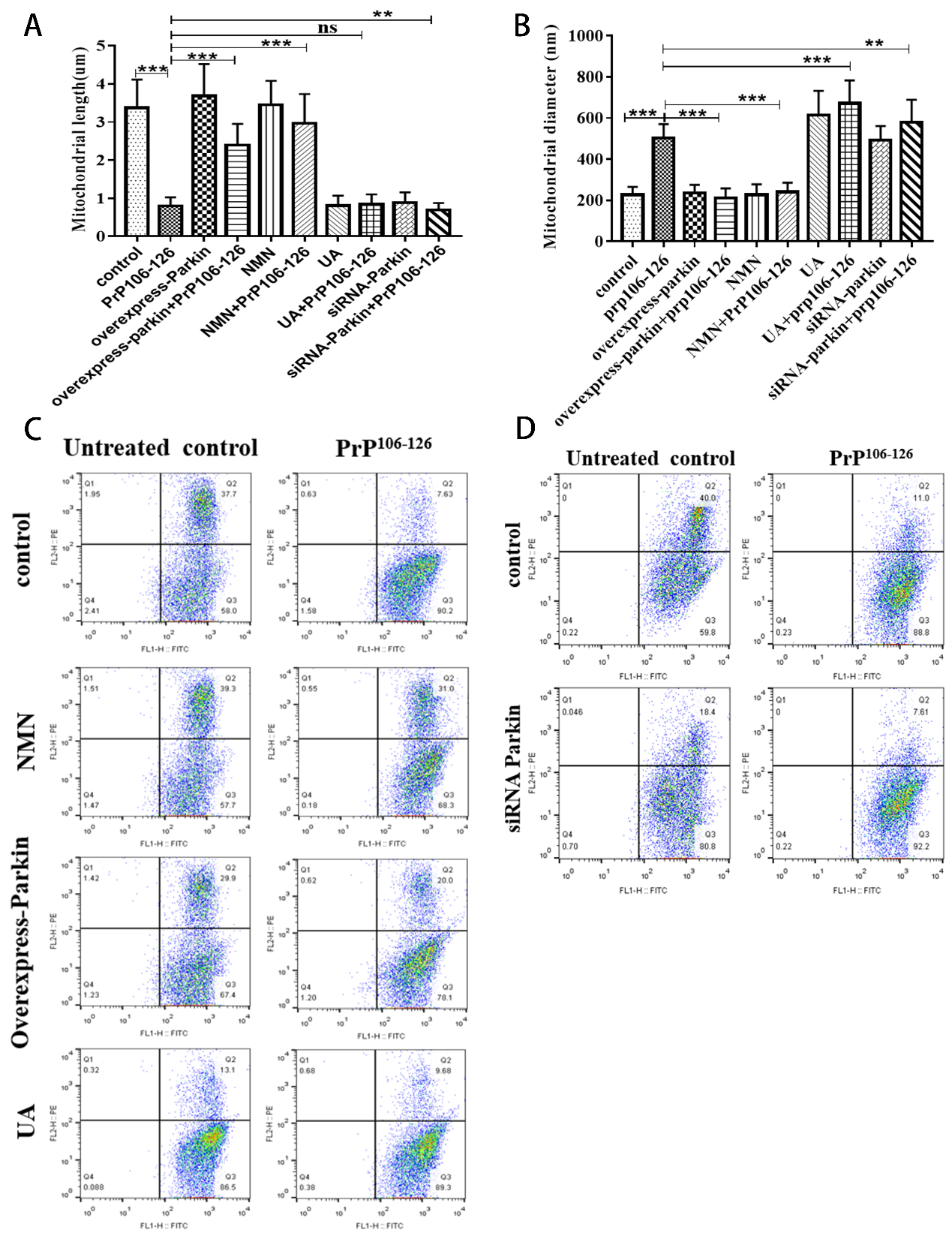


Figure S8. NMN supplementation and Parkin overexpression can alleviate PrP106-126-induced morphological mitochondrial damage and dysfunction. **(A)** Comparisons of the length of mitochondria in cells from **Figure 5A**. **(B)** Comparisons of the diameter of mitochondria in cells from **Figure 5B**. **(C, D)** FACS results of mitochondrial membrane potential (MMP) assays of N2a cells with overexpressed and knocked down (siRNA) parkin, and with and without PrP106-126, NMN, or UA treatment. The dye JC-1 shifts from red to green fluorescence intensity as MMP reduces. Data are mean (SD). **P* < 0.05; ***P* < 0.01; ****P* < 0.001. All experiments were repeated at least three times.

**Figure S9**


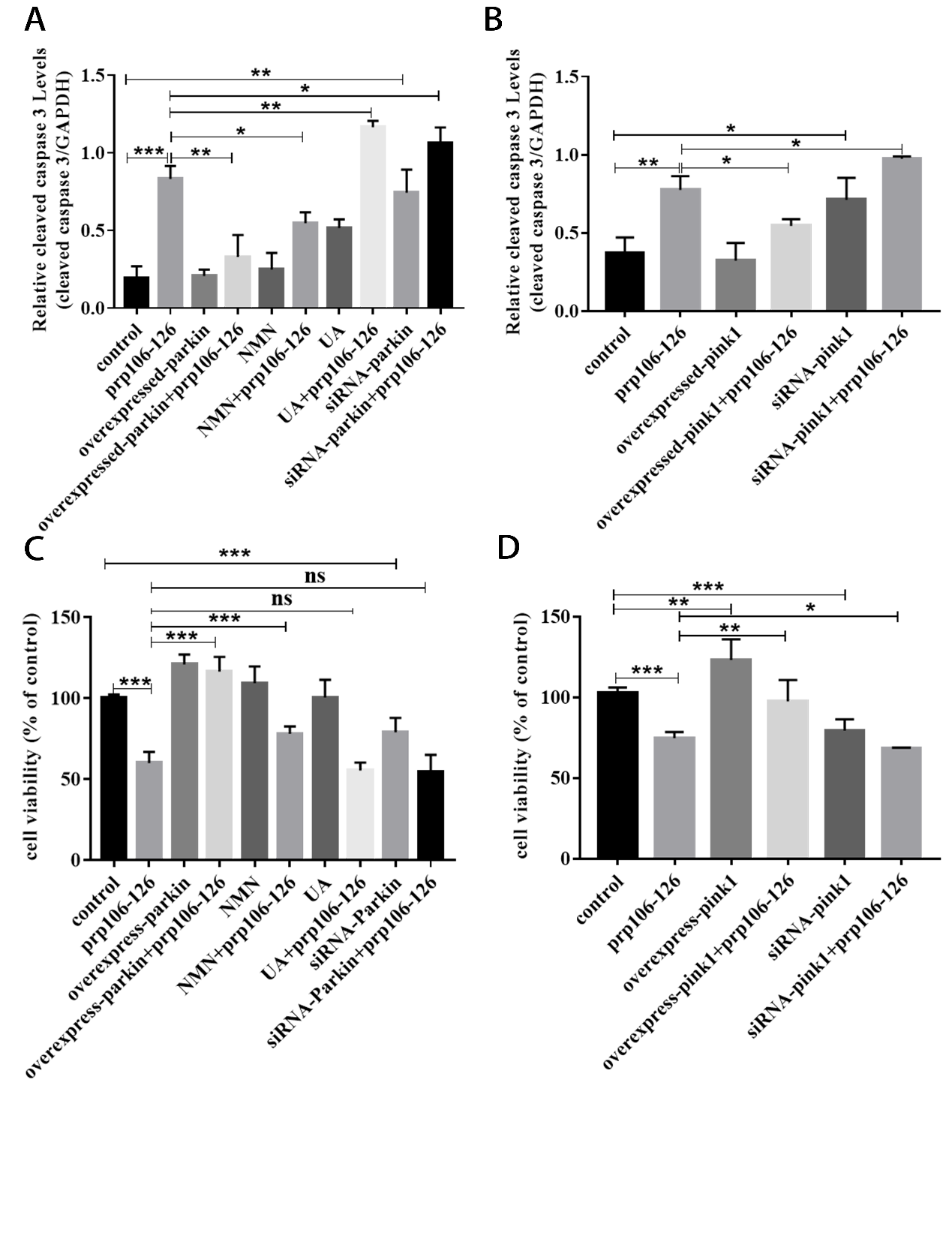


Figure S9. Activation of PINK1-parkin-mediated mitophagy attenuates PrP106-126-induced neuronal apoptosis. **(A, B)** Comparisons of cleaved caspase-3 protein levels, relative to controls (GAPDH), in the treated cells in **Figure 6E, F**. **(C, D)** Comparisons of cell activity detected by Cell Counting Kit-8 (CCK-8) assays of control and treated cells. Cell viability was expressed as percent of the untreated control. Data are mean (SD). **P* < 0.05; ***P* < 0.01; ****P* < 0.001. All experiments were repeated at least three times.
