# Supplementary material for: PINK1-parkin-mediated neuronal mitophagy deficiency in prion disease
Source: Cell Death Dis. 2022 Feb 18;13(2):162. doi: 10.1038/s41419-022-04613-2 (PMC8858315; doi:10.1038/s41419-022-04613-2)
Supplement: Supplementary file 2 — Original Data File [file 41419_2022_4613_MOESM2_ESM.docx]

**Figure 1**

**Figure 1D**




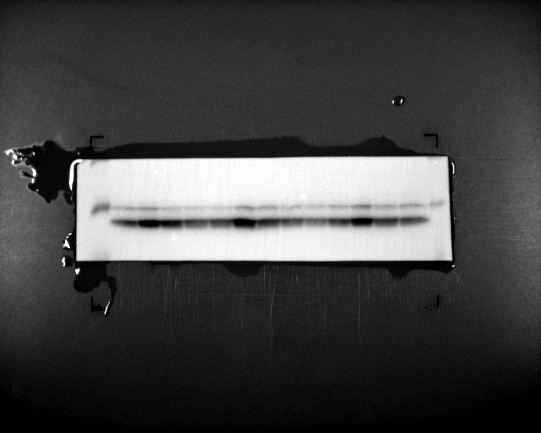


**LC3 LC3**




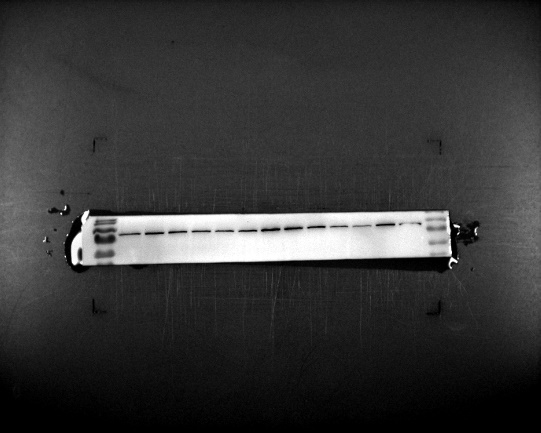


**P-TBK1 P-TBK1**




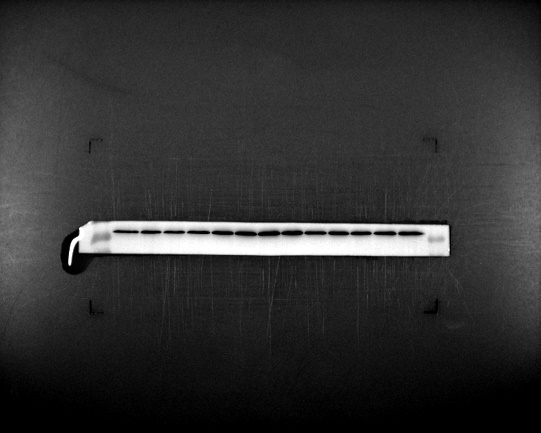


**GAPDH GAPDH**




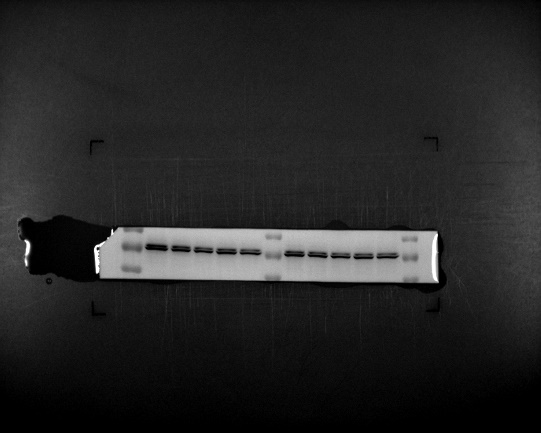


**OPTN OPTN**




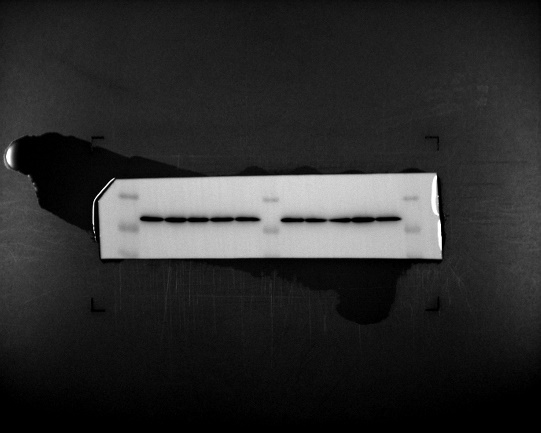


**GAPDH GAPDH**

**Figure 1E**




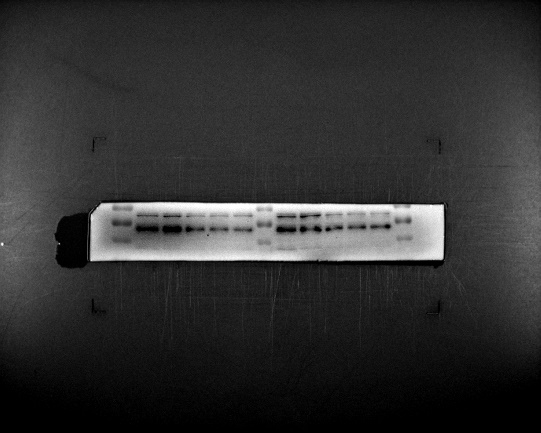


**PINK1 PINK1**




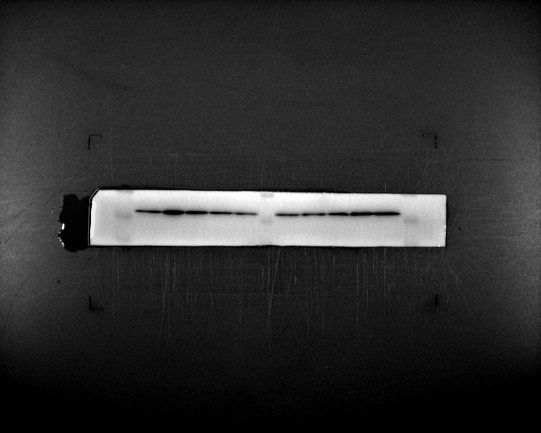


**GAPDH GAPDH**




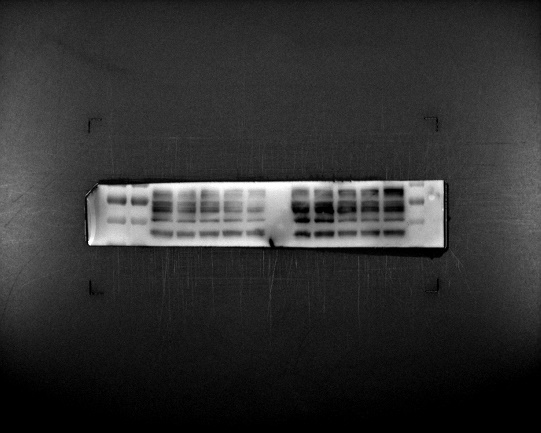


**Parkin Parkin**




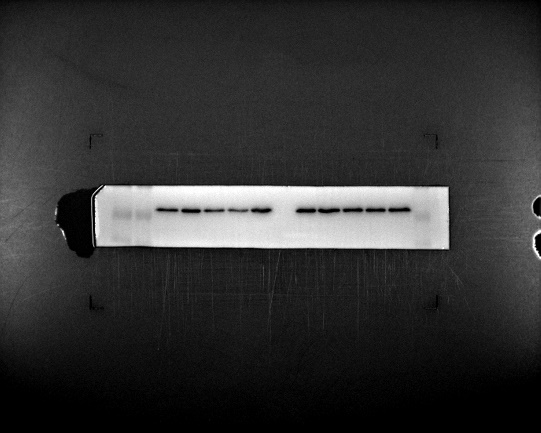


**GAPDH GAPDH**

**Figure 2**

**Figure 2F**




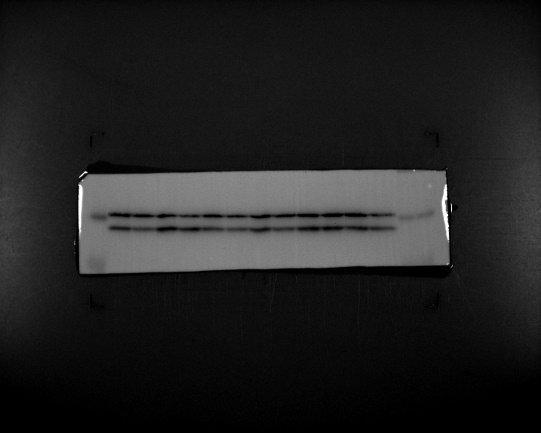


**LC3 LC3**




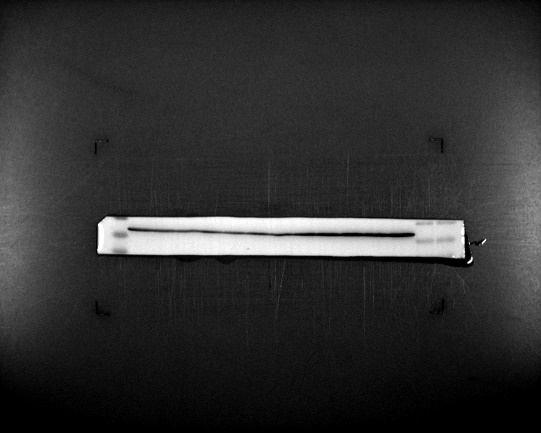


**GAPDH GAPDH**




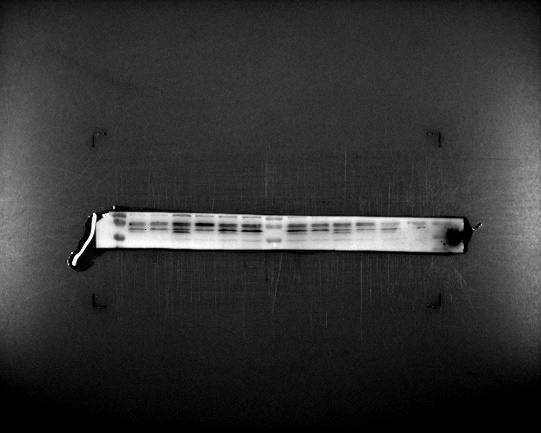


**OPTN OPTN**




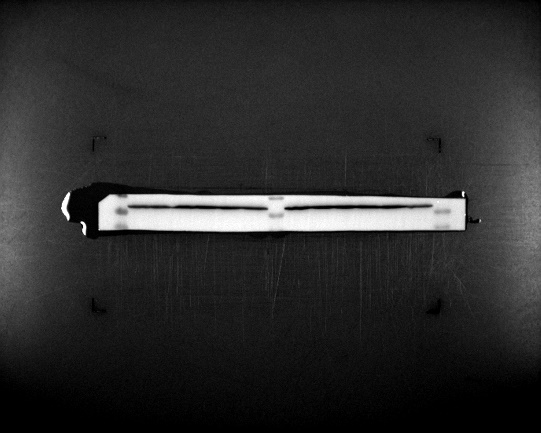


**GAPDH GAPDH**

**Figure 3**

**Figure 3A**




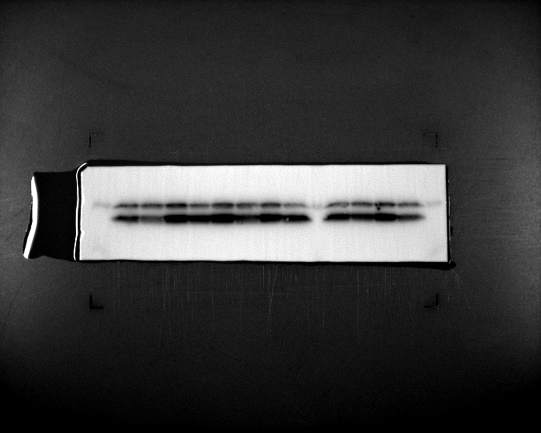


**LC3 LC3**




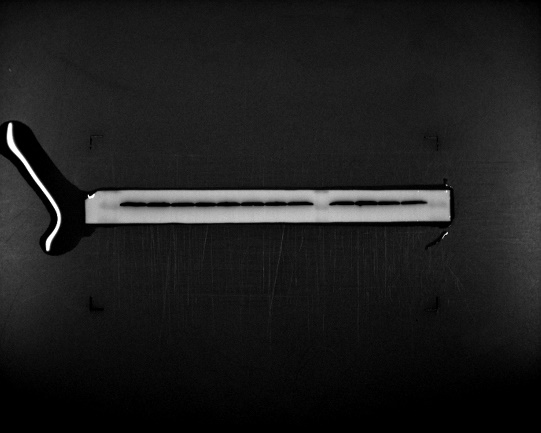


**GAPDH GAPDH**

**Figure 3B**




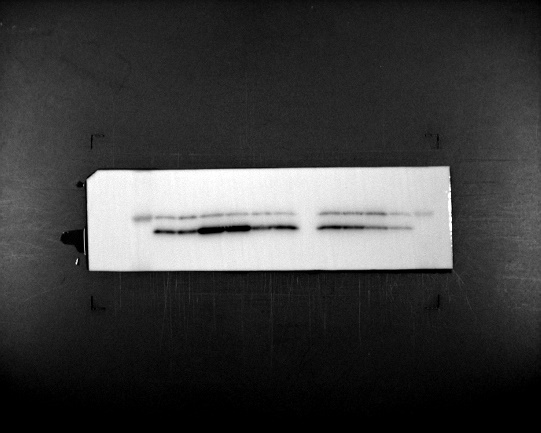


**LC3 LC3**




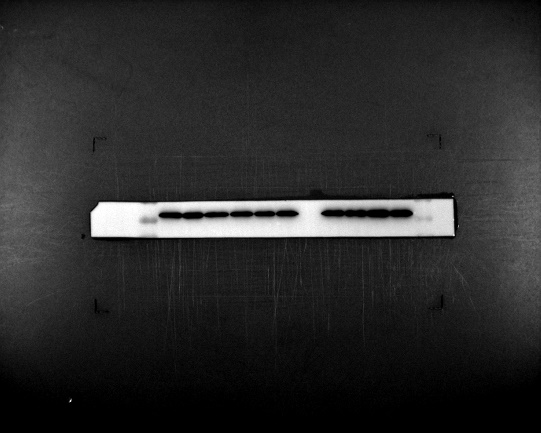


**GAPDH GAPDH**

**Figure 4**

**Figure 4A**




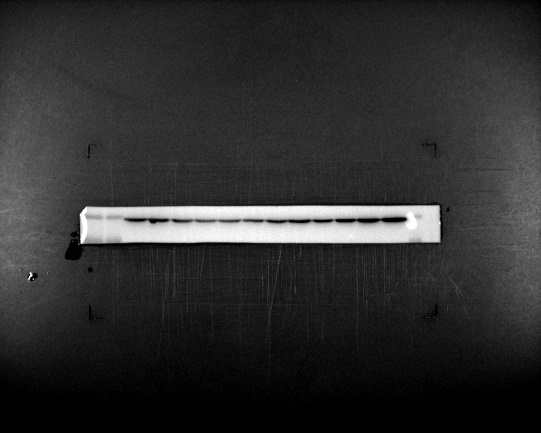


**TOMM40 TOMM40**




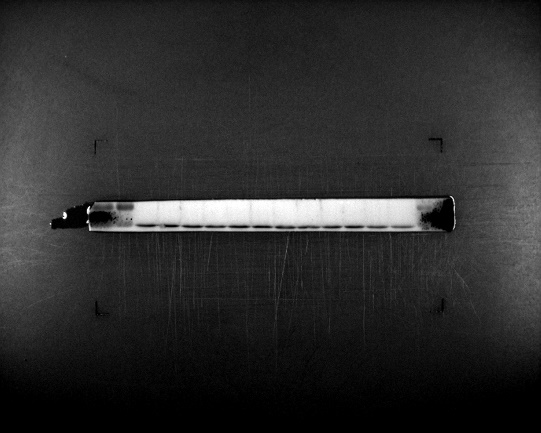


**TUBULIN TUBULIN**




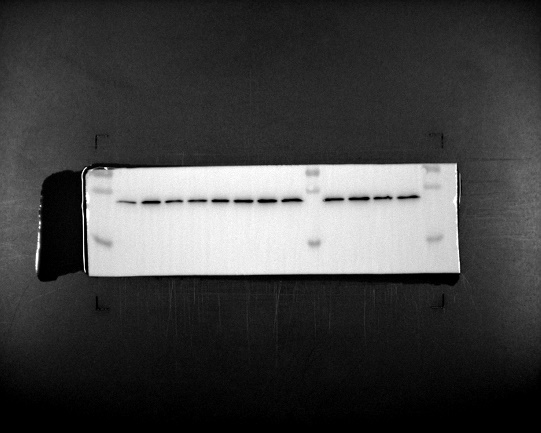


**SOD2 SOD2**




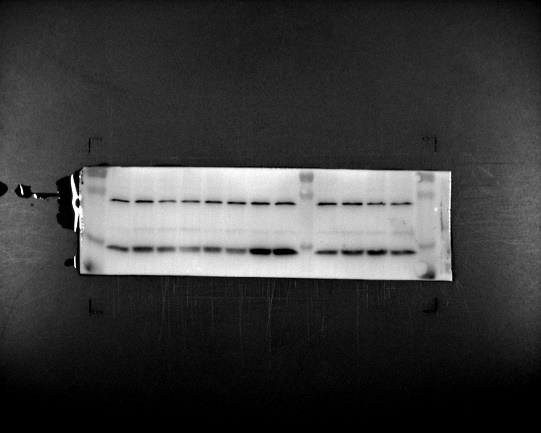


**COXIV COXIV**




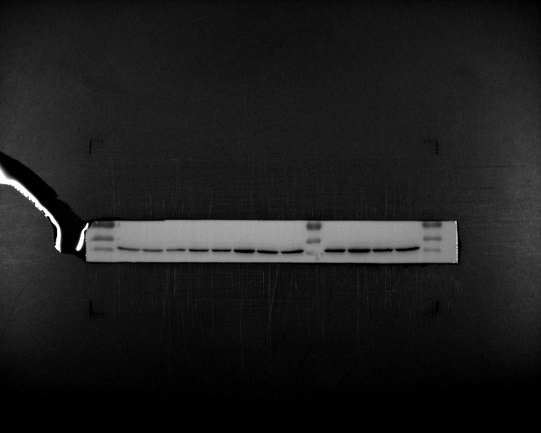


**β-ACTIN β-ACTIN**

**Figure 4B**




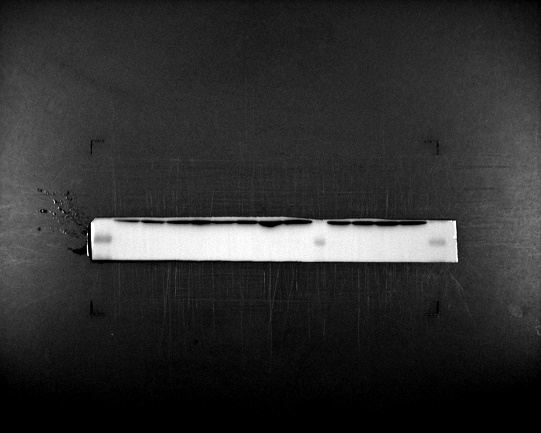


**TOMM40 TOMM40**




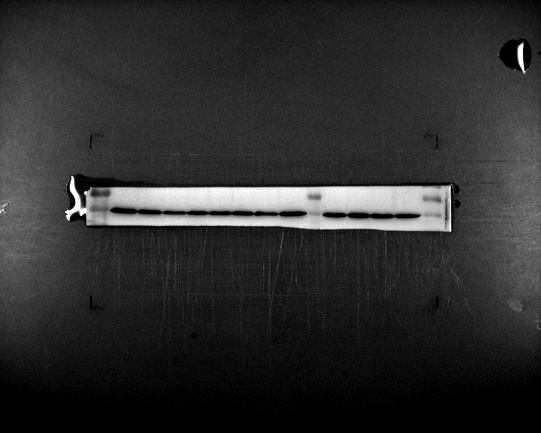


**TUBULIN TUBULIN**




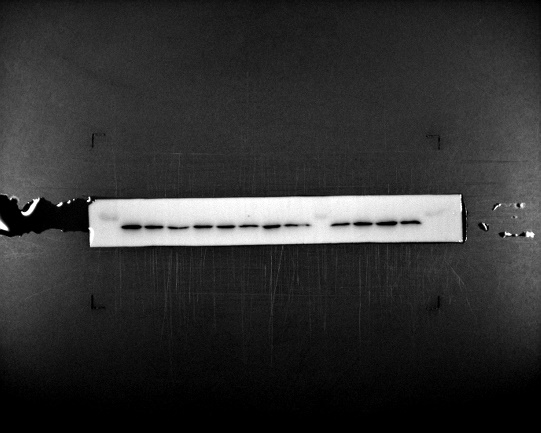


**SOD2 SOD2**




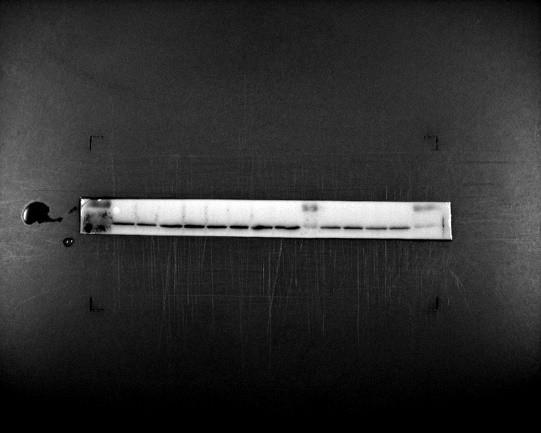


**TUBULIN TUBULIN**




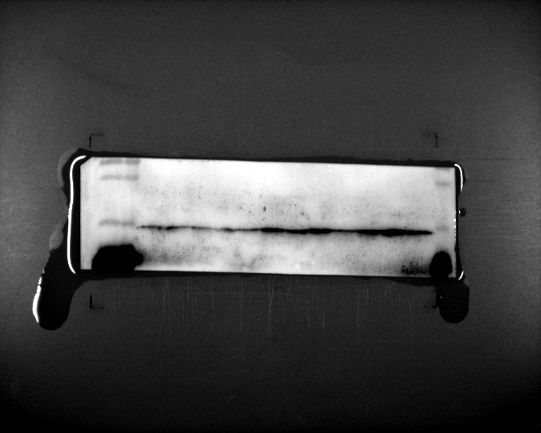


**COXIV COXIV**




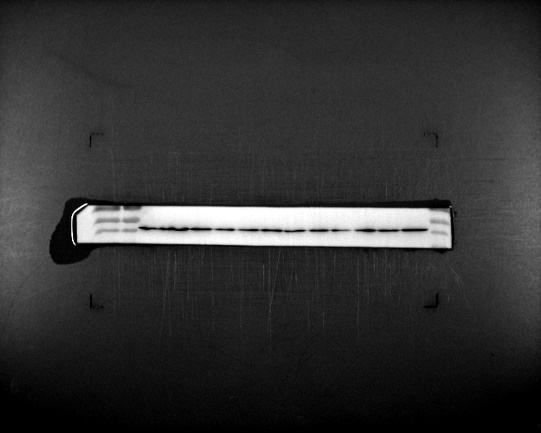


**β-ACTIN β-ACTIN**

**Figure 6**

**Figure 6E**




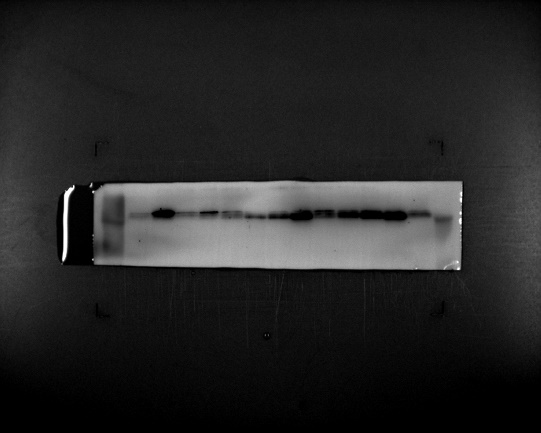


**Cleaved-Caspas3 Cleaved-Caspas3**




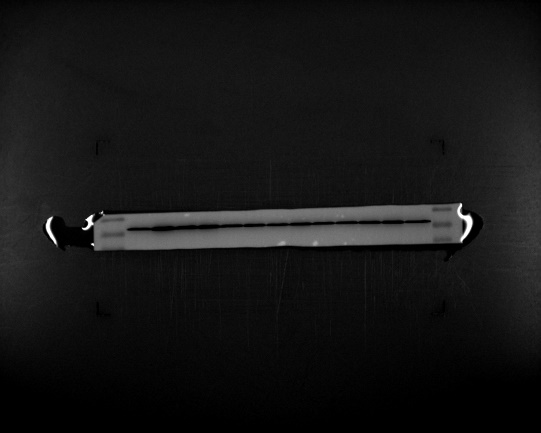


**GAPDH GAPDH**

**Figure 6F**




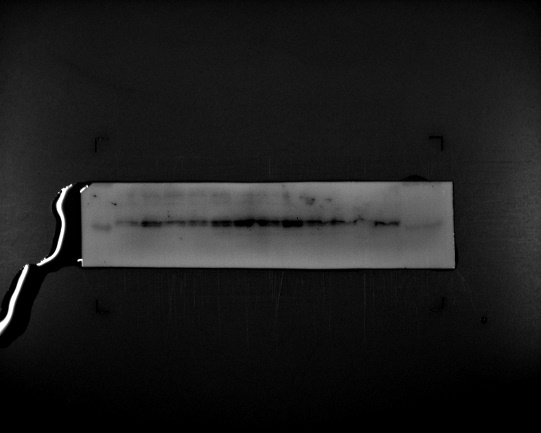


**Cleaved-Caspas3 Cleaved-Caspas3**




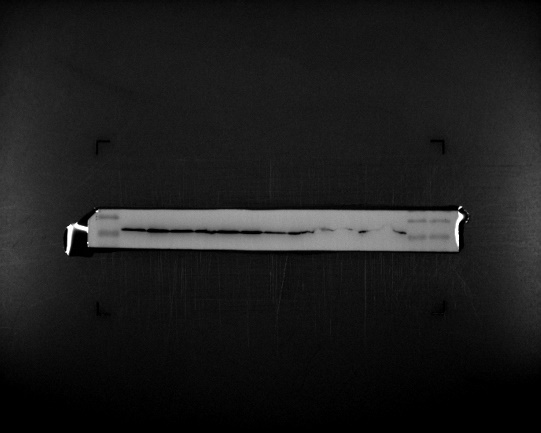


**GAPDH GAPDH**

**Figure S2**

**Figure S2A**




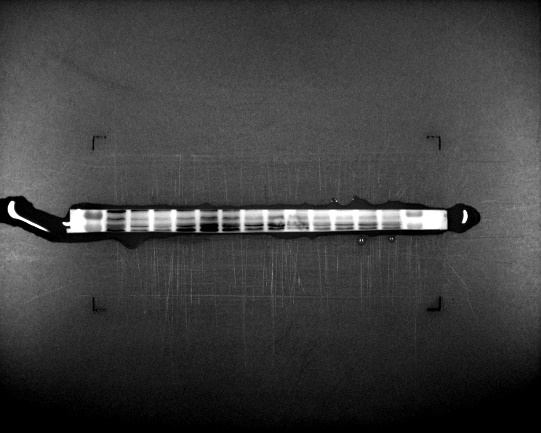


**PINK1 PINK1**




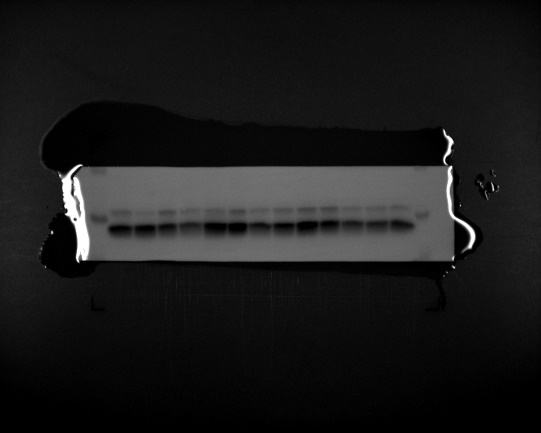


**LC3 LC3**




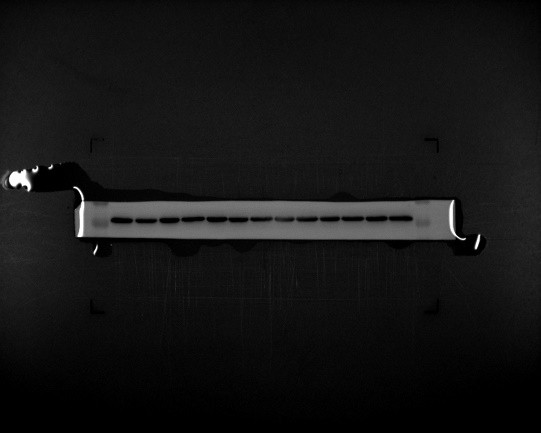


**GAPDH GAPDH**




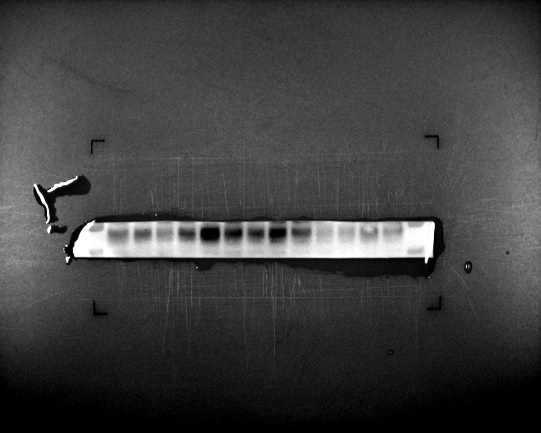


**Parkin Parkin**




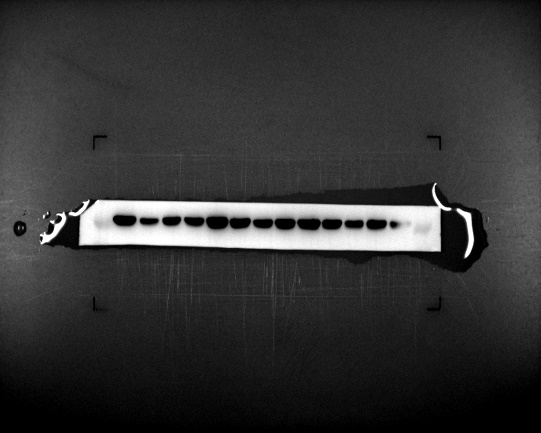


**GAPDH GAPDH**

**Figure S3**

**Figure S3A**




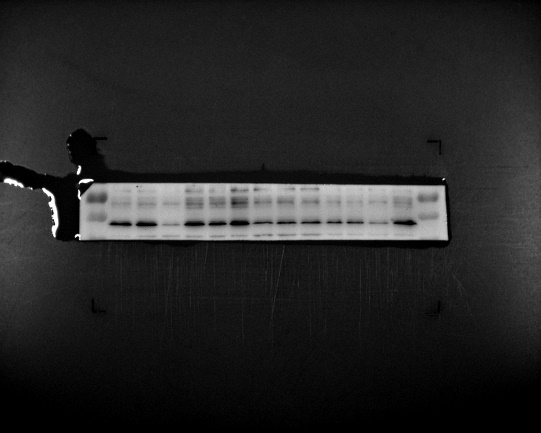


**PINK1 PINK1**




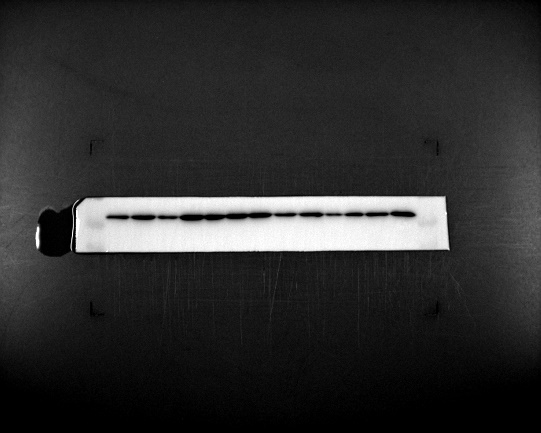


**GAPDH GAPDH**




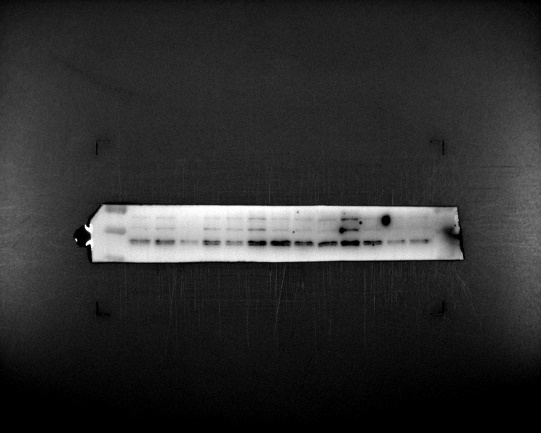


**Parkin Parkin**




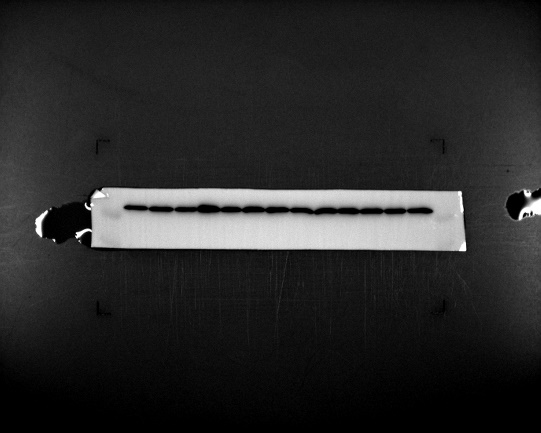


**GAPDH GAPDH**




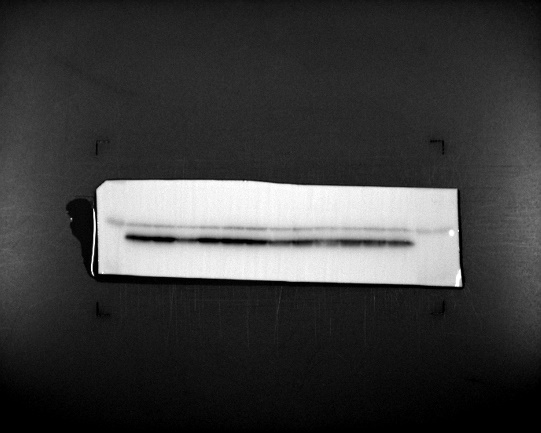


**LC3 LC3**




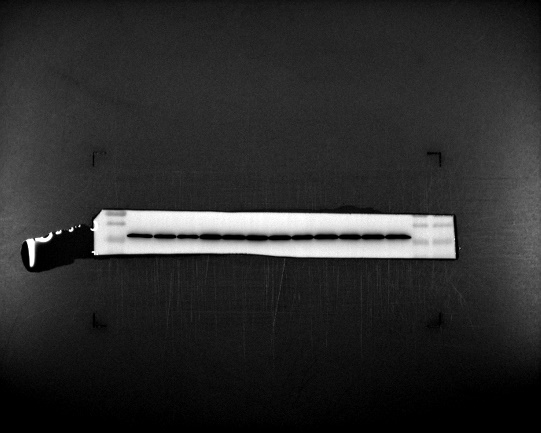


**GAPDH GAPDH**

**Figure S4**

**Figure S4A**




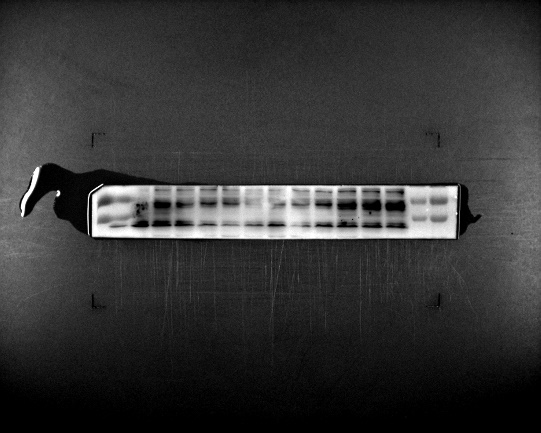


**PINK1 PINK1**




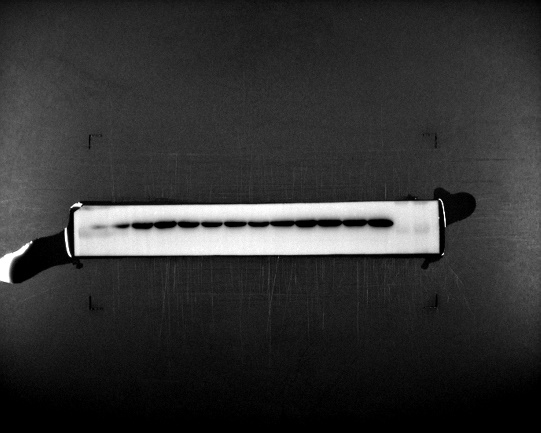


**GAPDH GAPDH**

**Figure S4C**




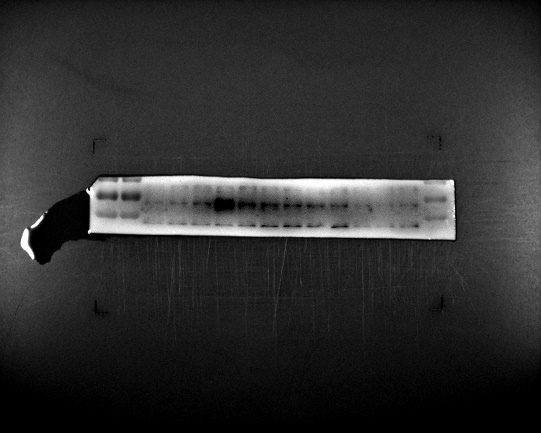


**PINK1 PINK1**




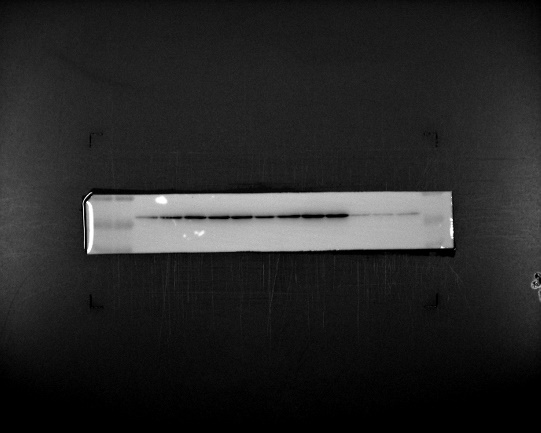


**GAPDH GAPDH**

**Figure S6**

**Figure S6A**




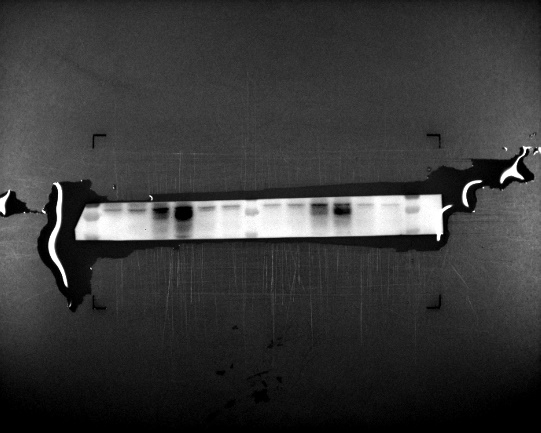


**Parkin Parkin**




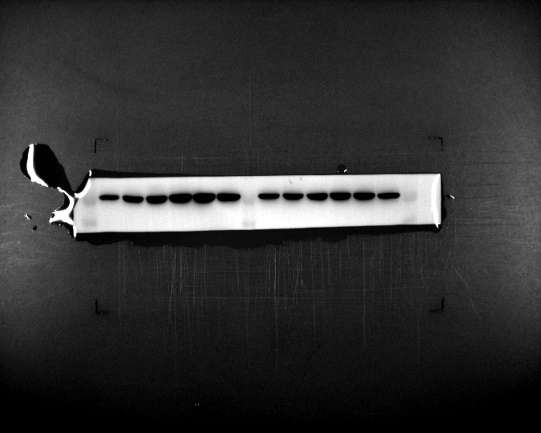


**GAPDH GAPDH**

**Figure S6C**




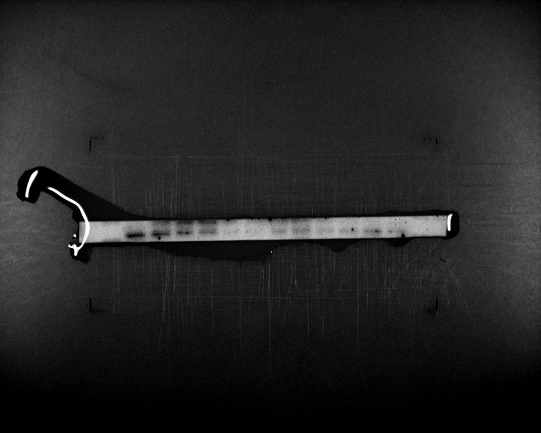


**Parkin Parkin**

**GAPDH GAPDH**
