# Supplementary material for: PINK1-parkin-mediated neuronal mitophagy deficiency in prion disease
Source: Cell Death Dis. 2022 Feb 18;13(2):162. doi: 10.1038/s41419-022-04613-2 (PMC8858315; doi:10.1038/s41419-022-04613-2)
Supplement: Supplementary file 3 — Original Data File [file 41419_2022_4613_MOESM3_ESM.docx]

**Figure 1**

**Figure 1A**


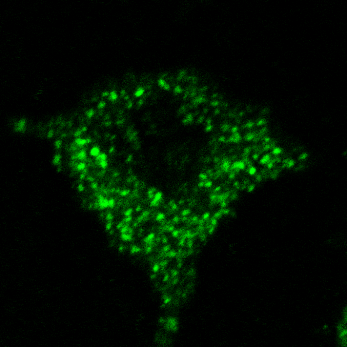

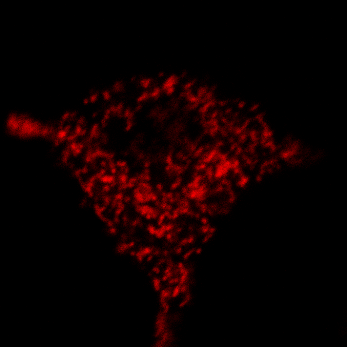

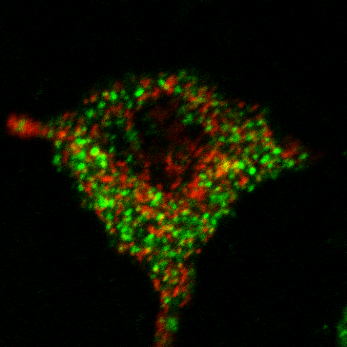


**Control**


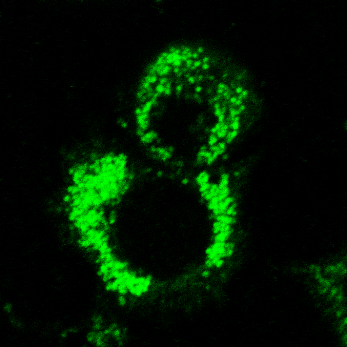

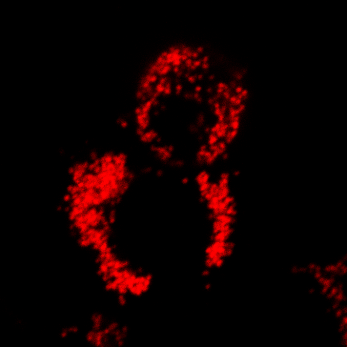

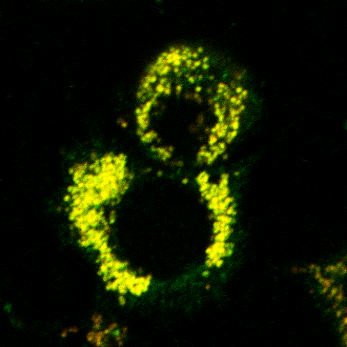


**PrP106-126 6h**


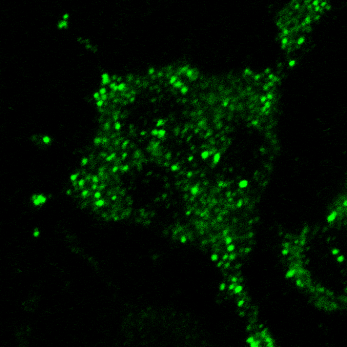

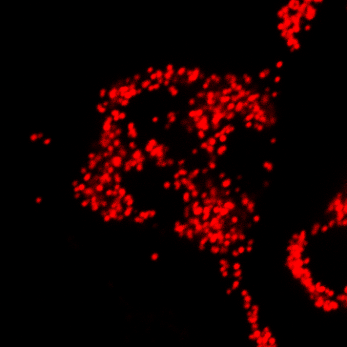

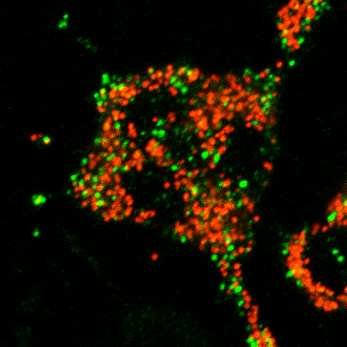


**PrP106-126 12h**


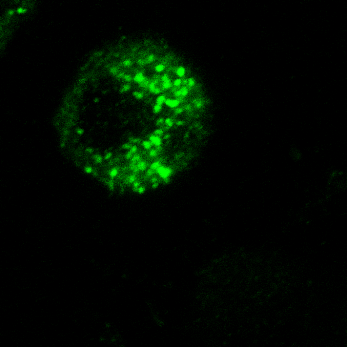

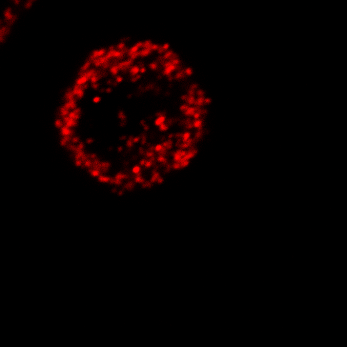

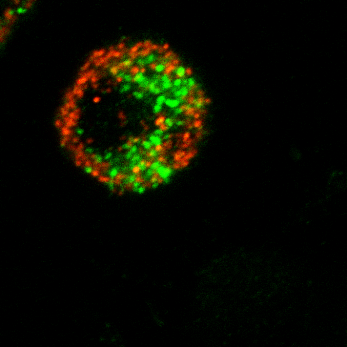


**PrP106-126 24h**


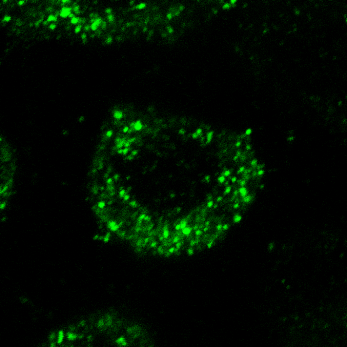

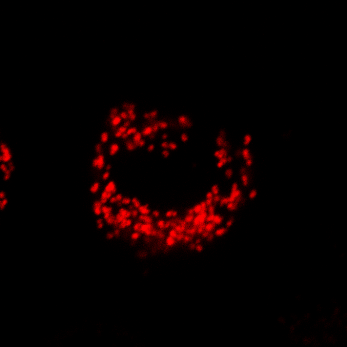

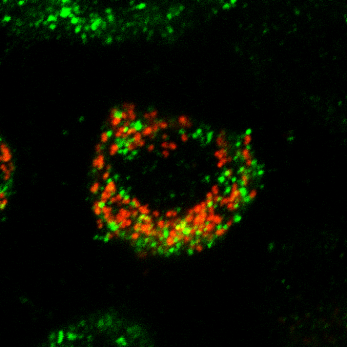


**PrP106-126 36h**

**Figure 2**

**Figure 2A**


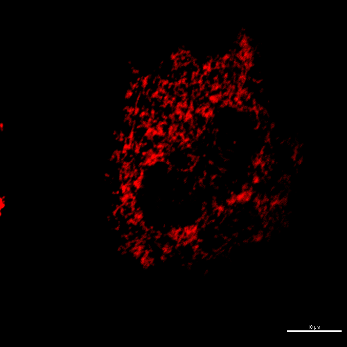

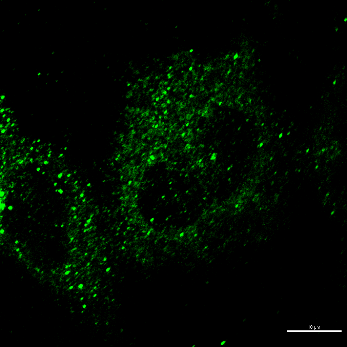

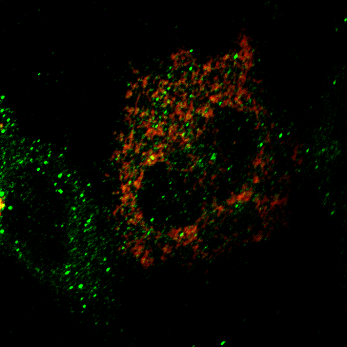


**Control**


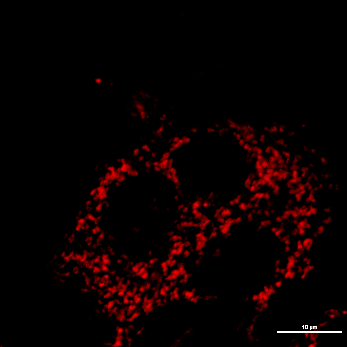

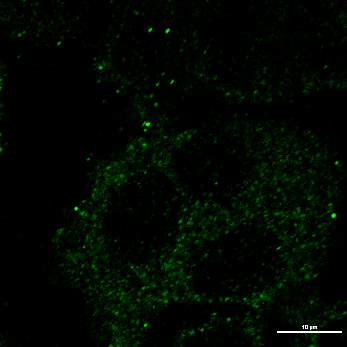

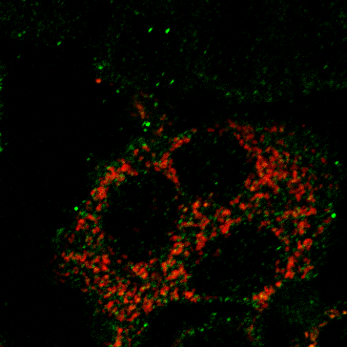


**PrP106-126 24h**


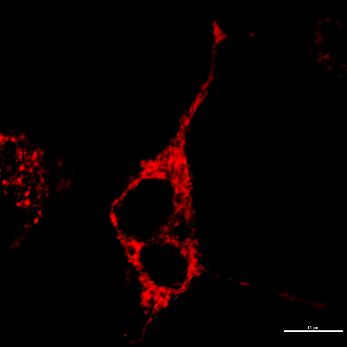

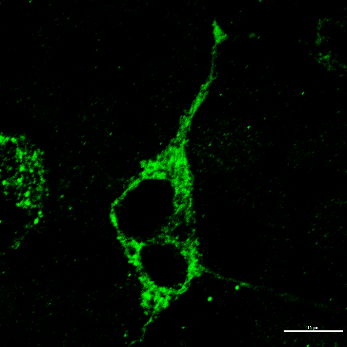

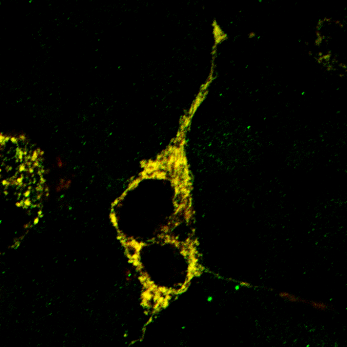


**Overexpress pink1**


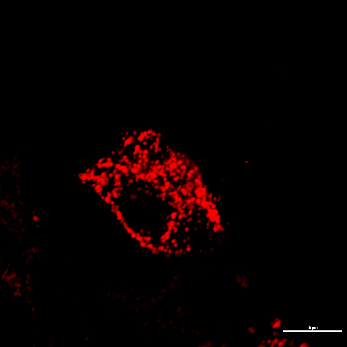

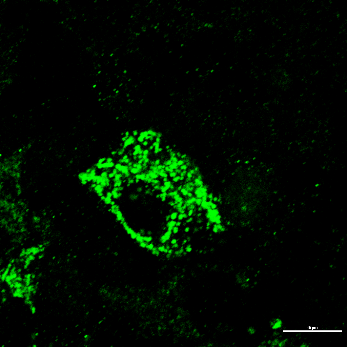

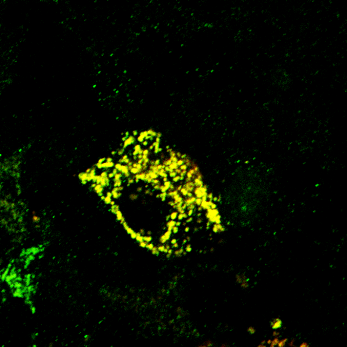


**Overexpress pink1+PrP**


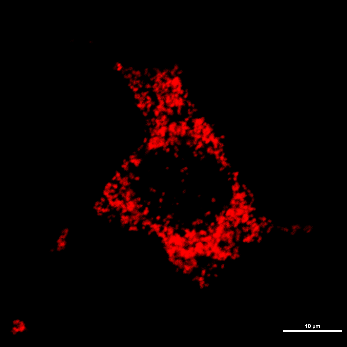

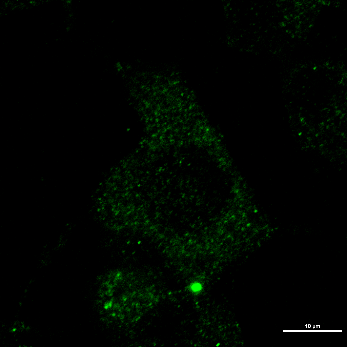

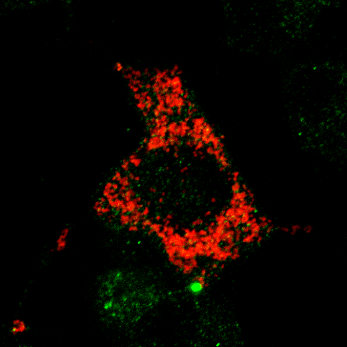


**siRNA pink1**


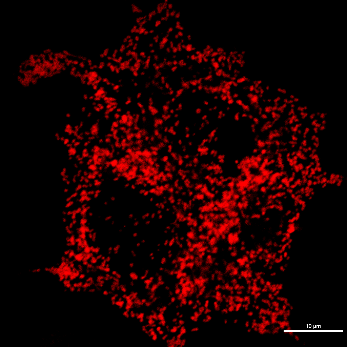

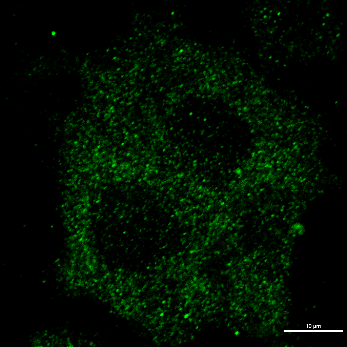

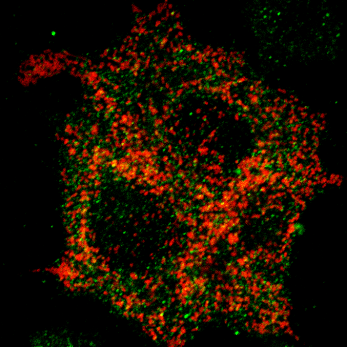


**siRNA pink1+PrP**

**Figure 2C**


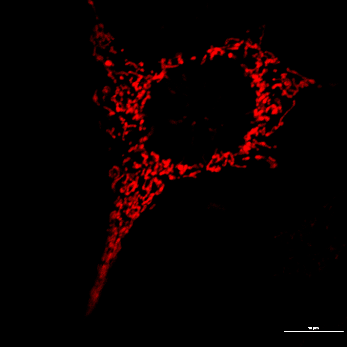

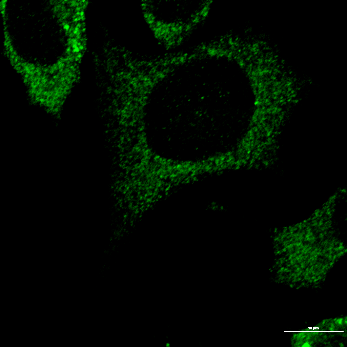

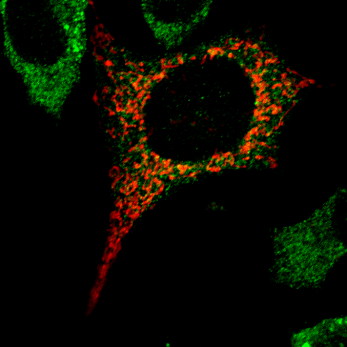


**Control**


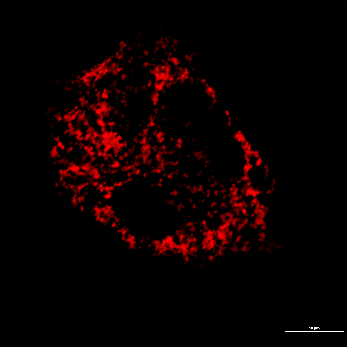

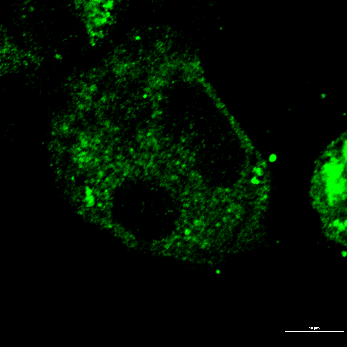

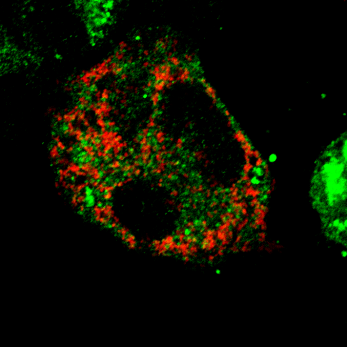


**PrP106-126 24h**


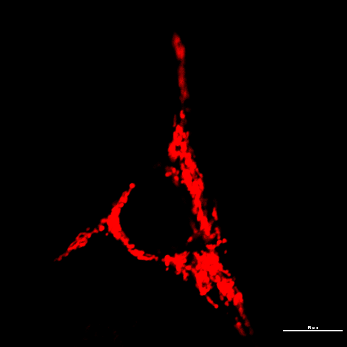

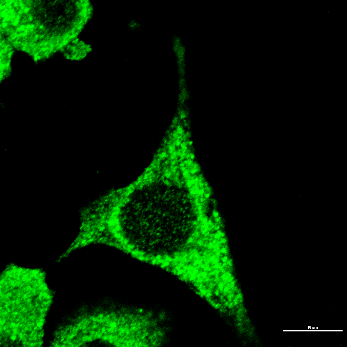

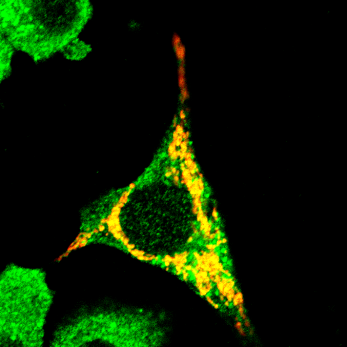


**Overexpress pink1**


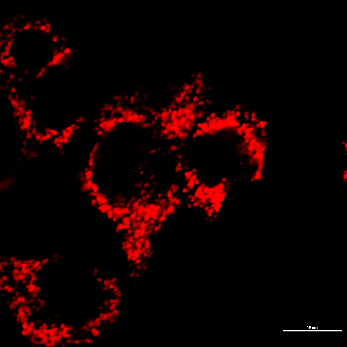

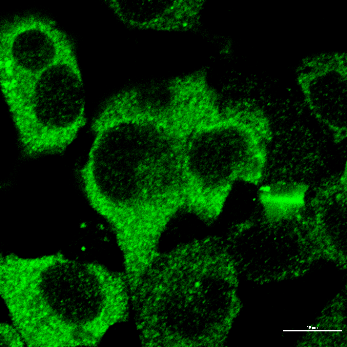

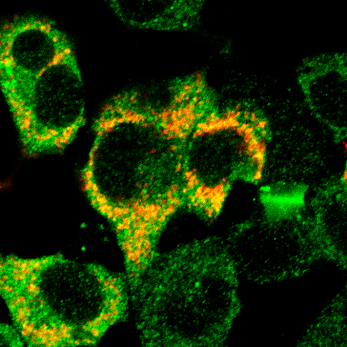


**Overexpress pink1+PrP**


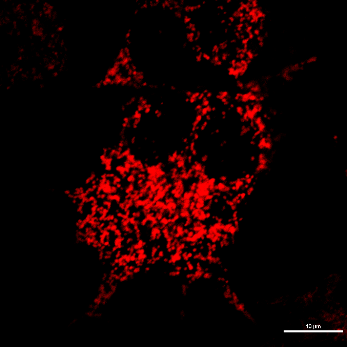

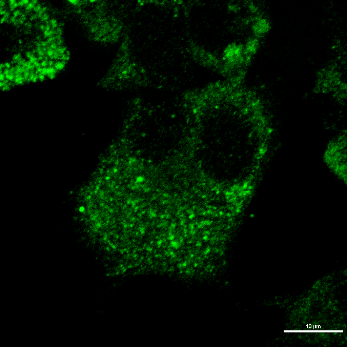

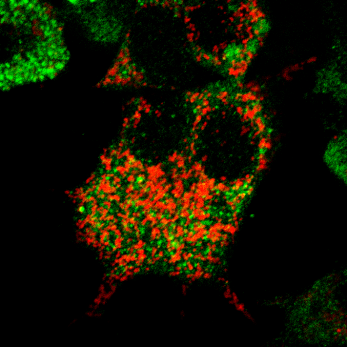


**siRNA pink1**


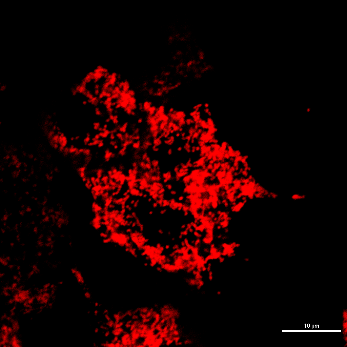

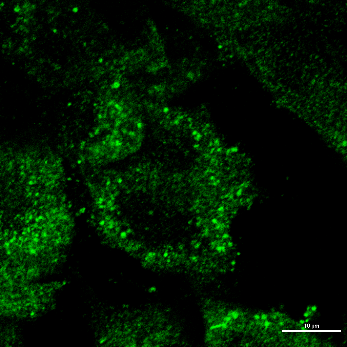

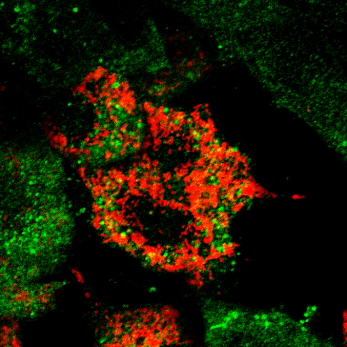


**siRNA pink1+PrP**

**Figure 3**

**Figure 3C**


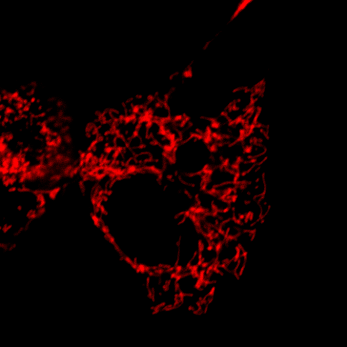

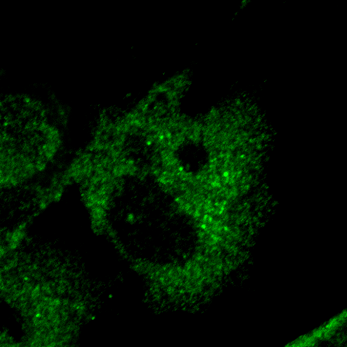

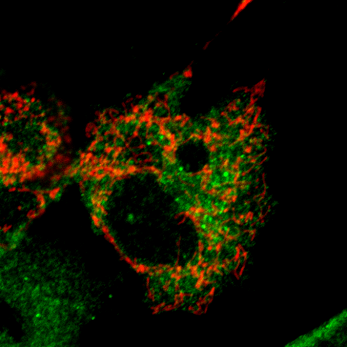


**Control**


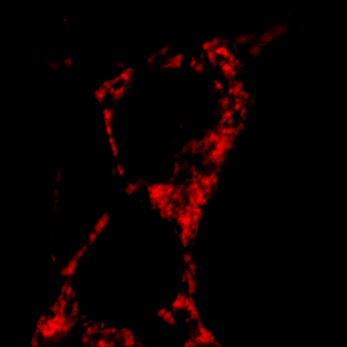

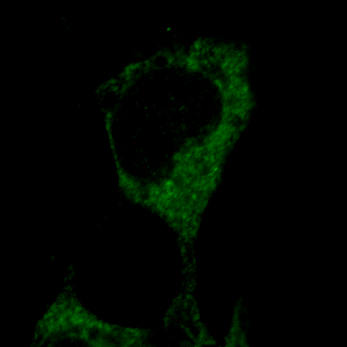

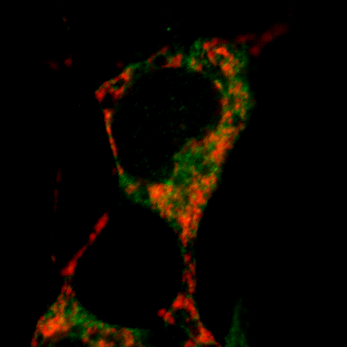


**PrP106-126 24h**


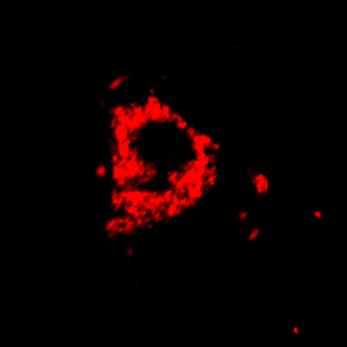

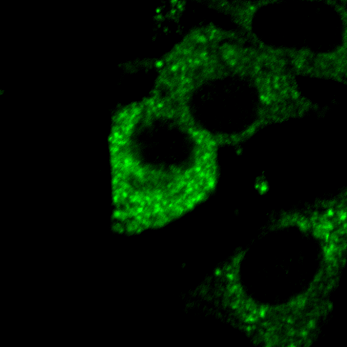

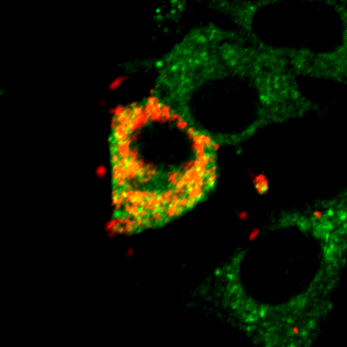


**Overexpress parkin**


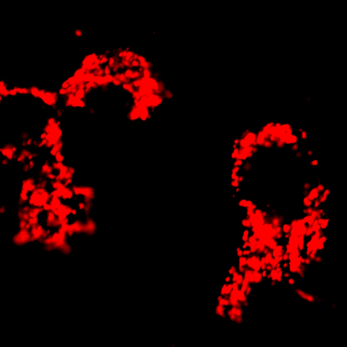

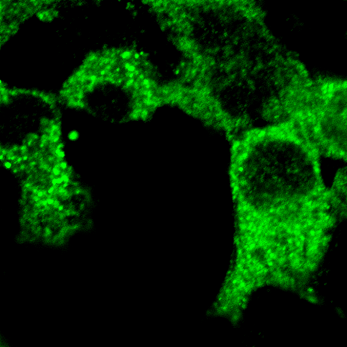

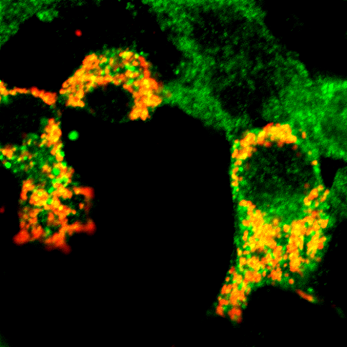


**Overexpress parkin+PrP**


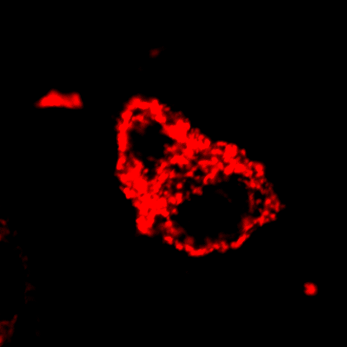

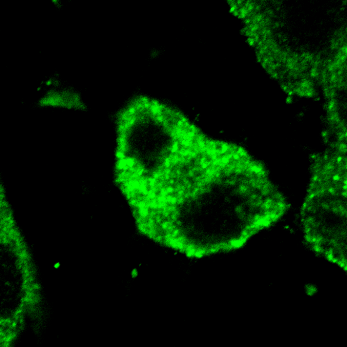

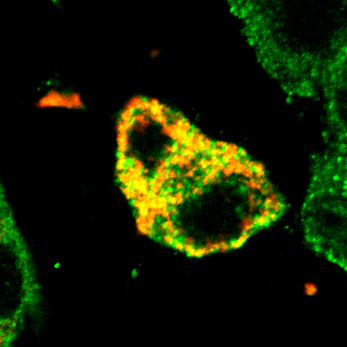


**NMN**


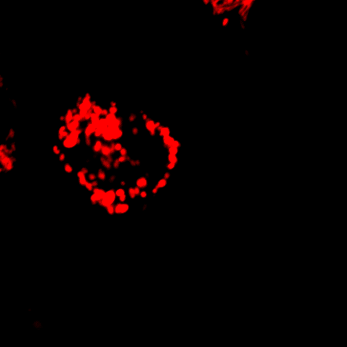

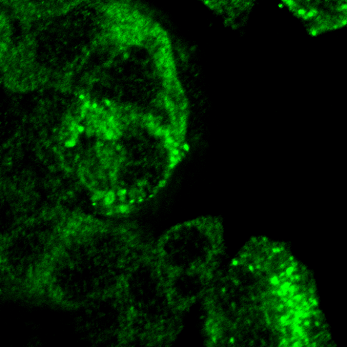

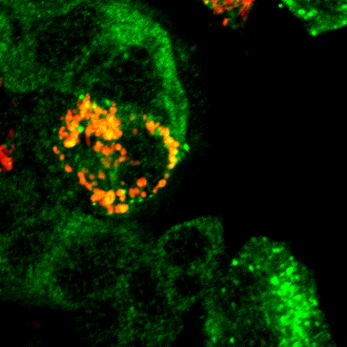


**NMN+PrP**


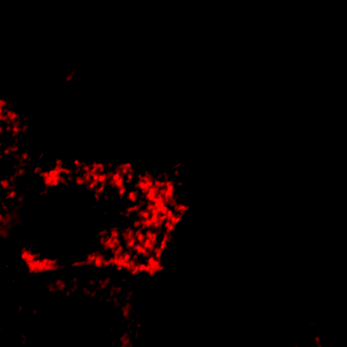

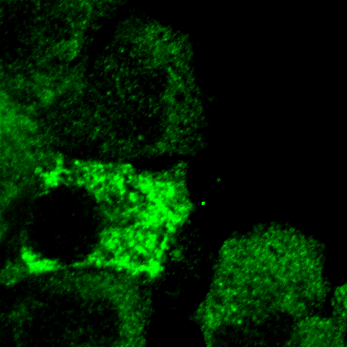

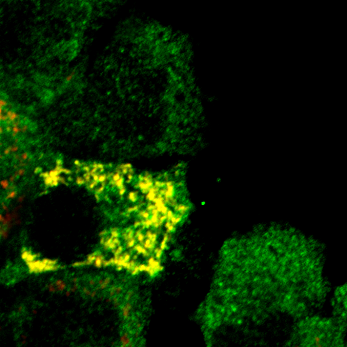


**UA**


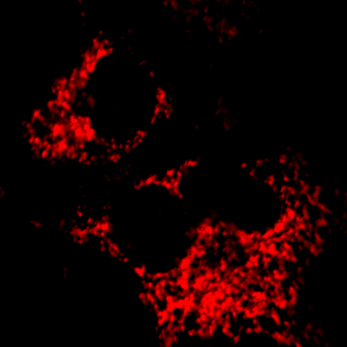

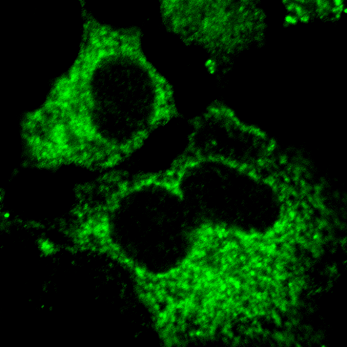

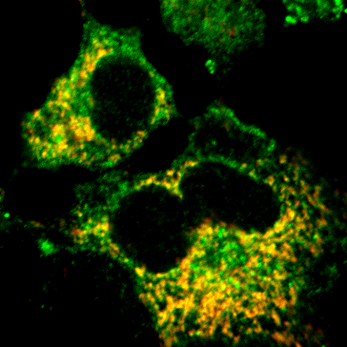


**UA+PrP**


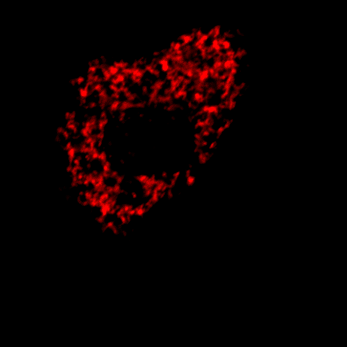

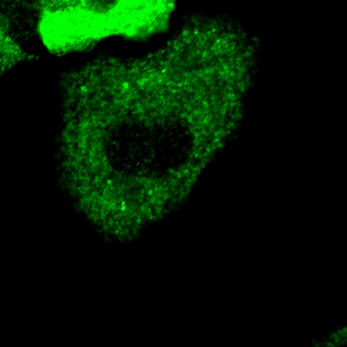

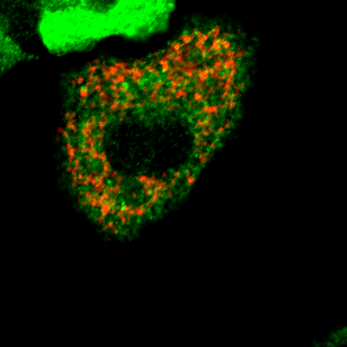


**siRNA parkin**


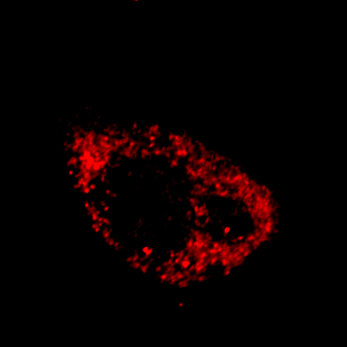

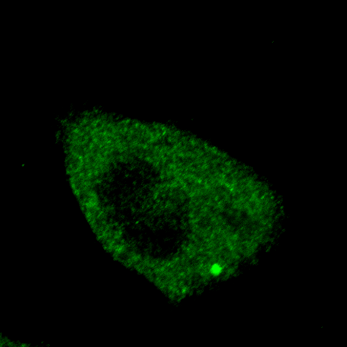

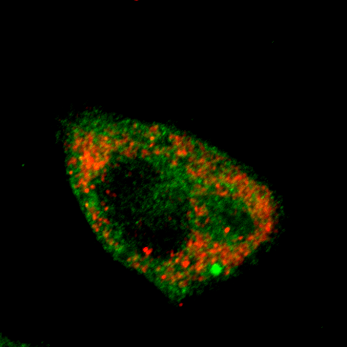


**siRNA parkin+PrP**

**Figure 5**

**Figure 5A**


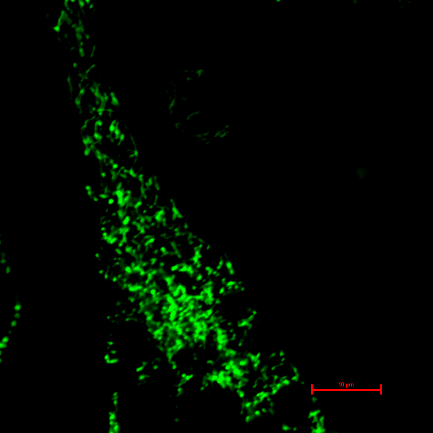

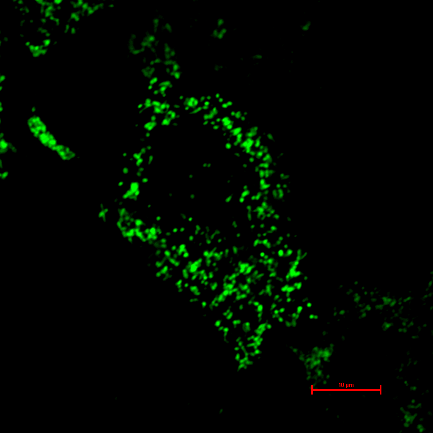


**Control PrP106-126 24 h**


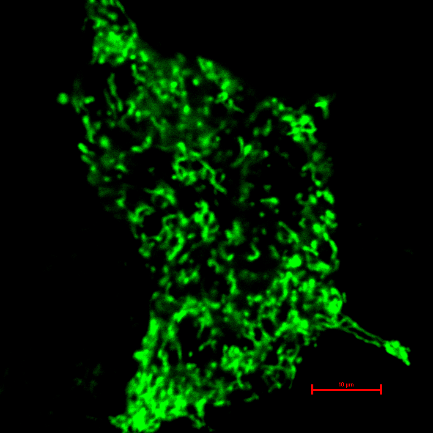

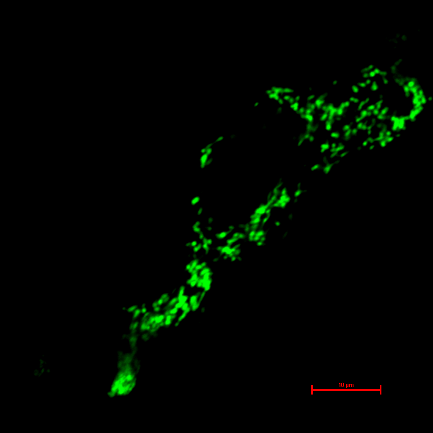


**Overexpress parkin Overexpress parkin+PrP**


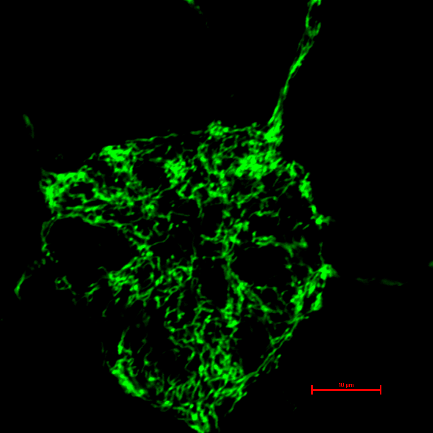

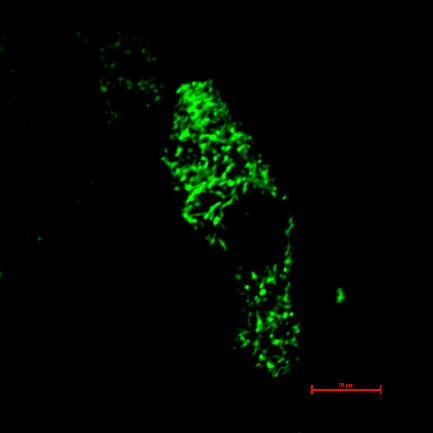


**NMN NMN+PrP**


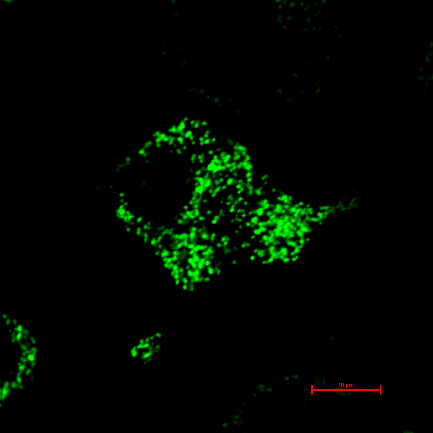

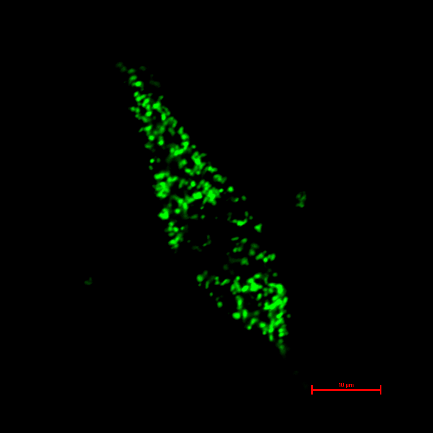


**UA UA+PrP**


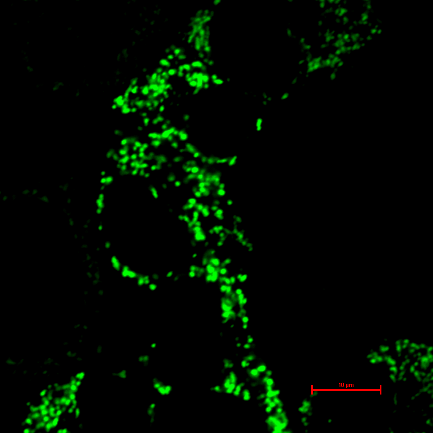

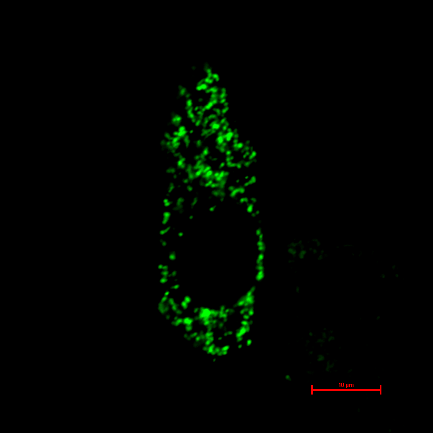


**siRNA parkin siRNA parkin+PrP**

**Figure 5B**


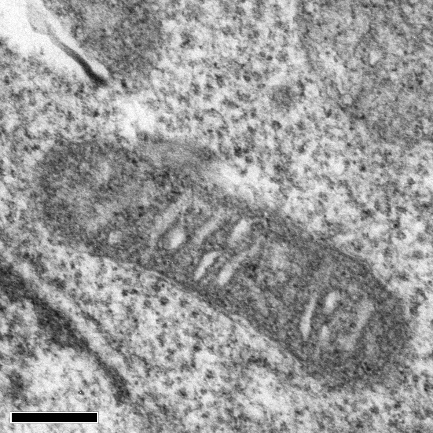

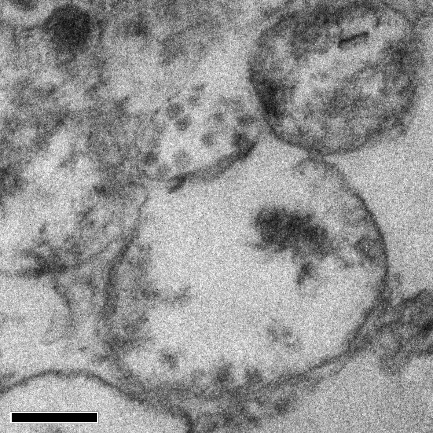


**Control PrP106-126 24 h**


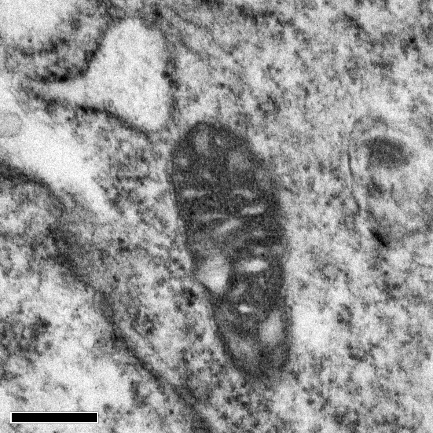

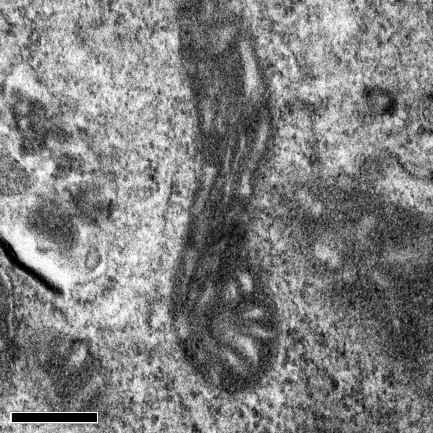


**Overexpress parkin Overexpress parkin+PrP**


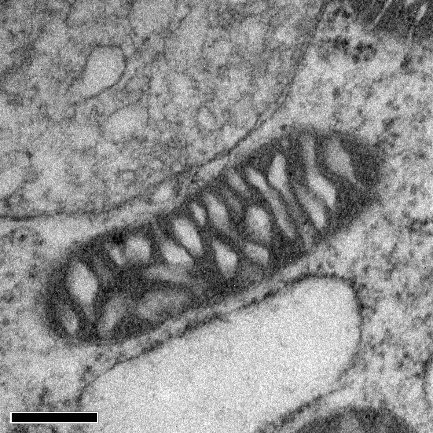

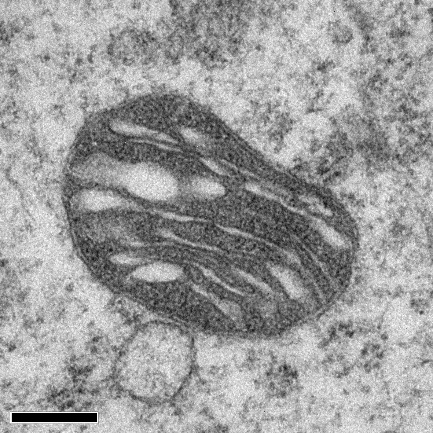


**NMN NMN+PrP**


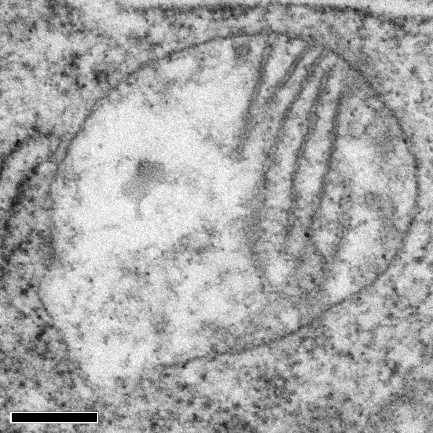

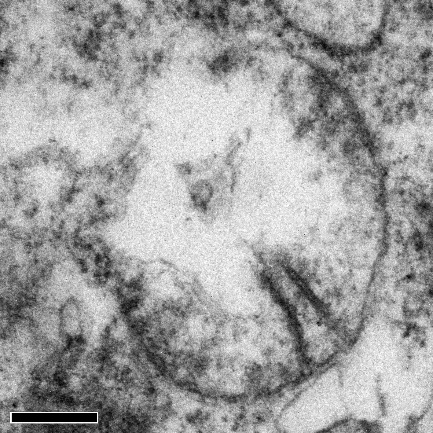


**UA UA+PrP**


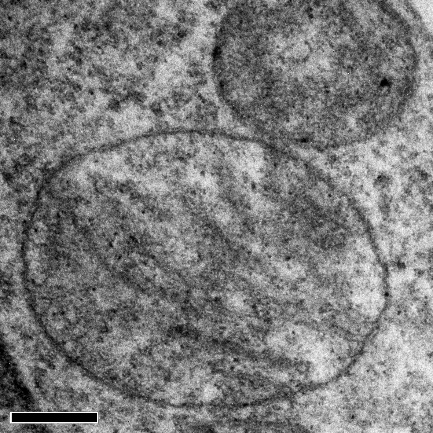


**siRNA parkin siRNA parkin+PrP**

**Figure 6**

**Figure 6A**

**Control**

**PrP106-126 24 h**

**Overexpress parkin**

**Overexpress parkin+PrP**

**NMN**

**NMN+PrP**

**UA**

**UA+PrP**

**siRNA parkin**

**siRNA parkin+PrP**

**Figure 6C**

**Control**

**PrP106-126 24 h**

**Overexpress pink1**

**Overexpress pink1+PrP**

**siRNA pink1**

**siRNA pink1+PrP**
