# Supplementary material for: PINK1-parkin-mediated neuronal mitophagy deficiency in prion disease
Source: Cell Death Dis. 2022 Feb 18;13(2):162. doi: 10.1038/s41419-022-04613-2 (PMC8858315; doi:10.1038/s41419-022-04613-2)
Supplement: Supplementary file 4 — Original Data File [file 41419_2022_4613_MOESM4_ESM.docx]

**Figure 1**

**Figure 1C**


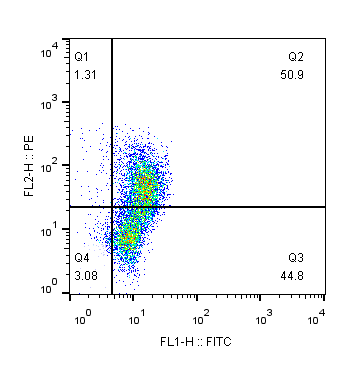


**Control**

**
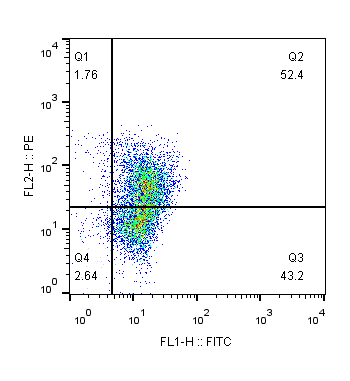
**

**PrP106-126 6h**


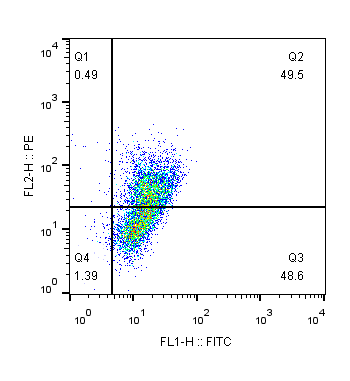


**PrP106-126 12h**

**
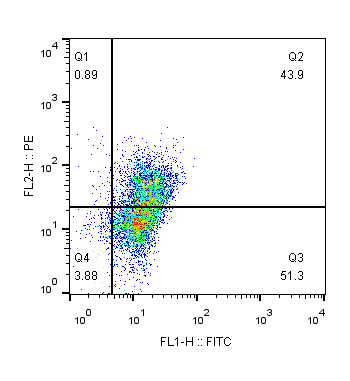
**

**PrP106-126 24h**

**
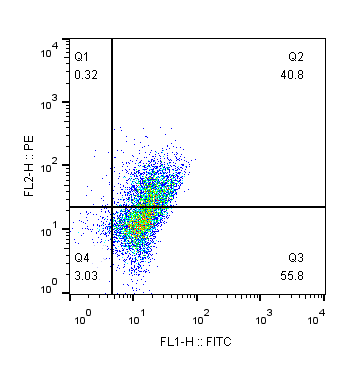
**

**PrP106-126 36h**

**Figure 2**

**Figure 2E**


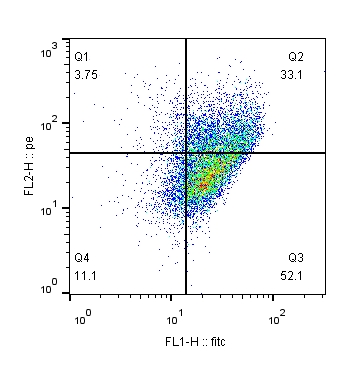


**Control**

**
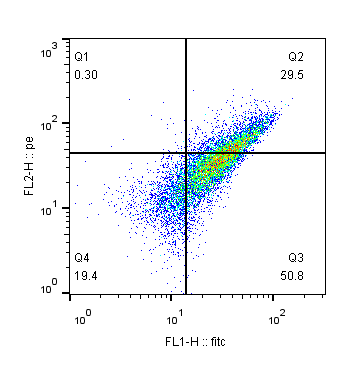
**

**PrP106-126 24h**

**
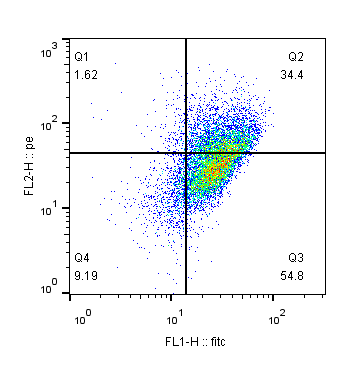
**

**Overexpress pink1**

**
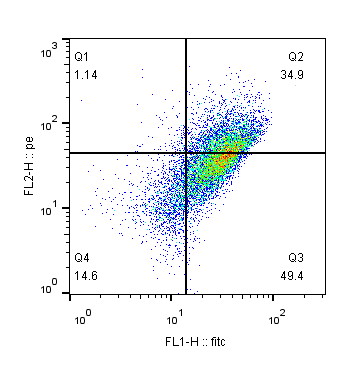
**

**Overexpress pink1+prp**

**
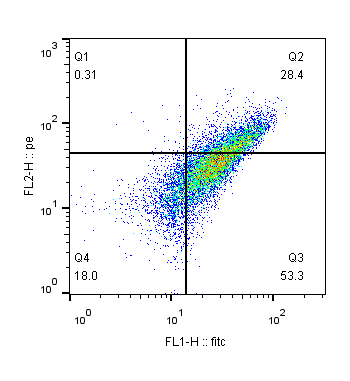
**

**siRNA pink1**

**
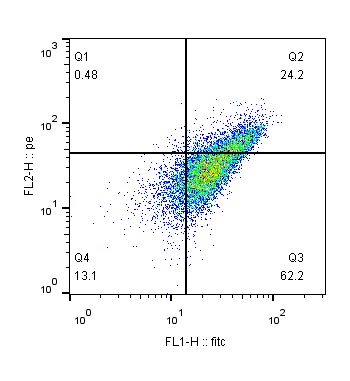
**

**siRNA pink1+prp**

**Figure 3**

**Figure 3E**

**
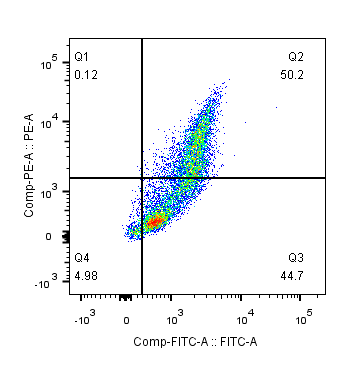
**

**Control**

**
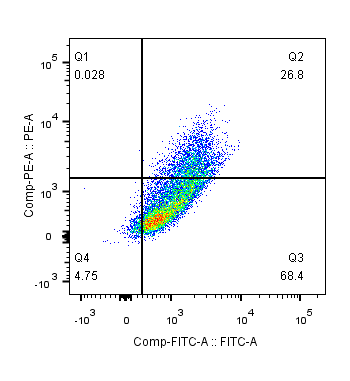
**

**PrP106-126 24h**

**
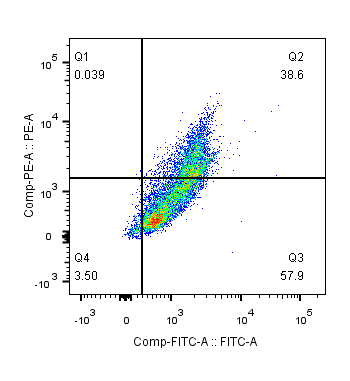
**

**Overexpress parkin**

**
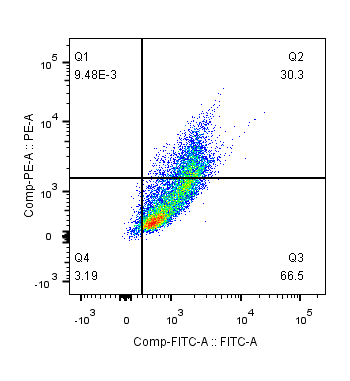
**

**Overexpress parkin+prp**

**
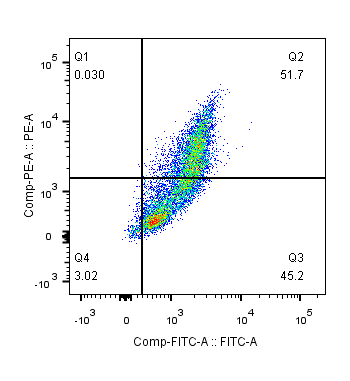
**

**NMN**

**
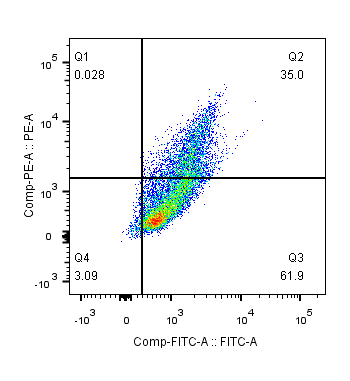
**

**NMN+prp**

**
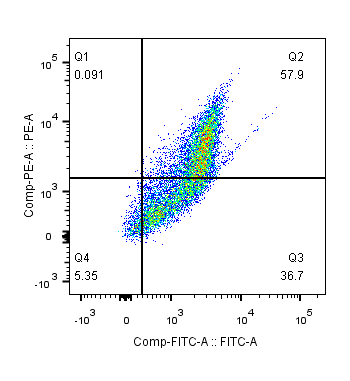
**

**UA**

**
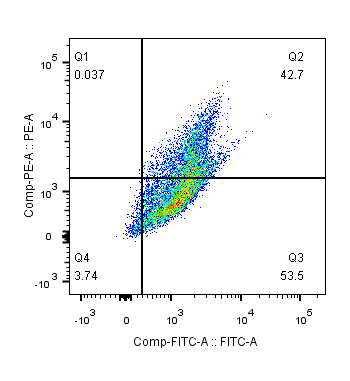
**

**UA+prp**

**Figure 3F**


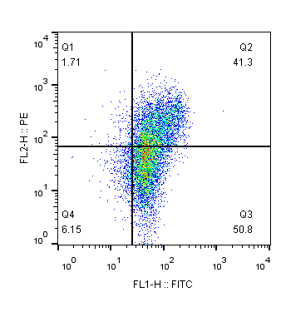


**Control**


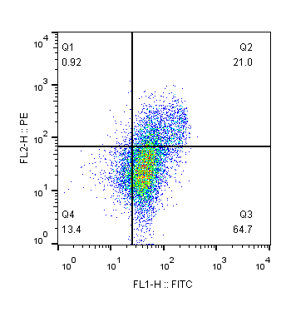


**PrP106-126 24h**


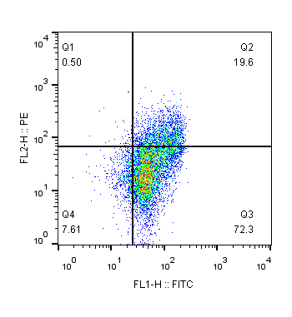


**siRNA parkin**


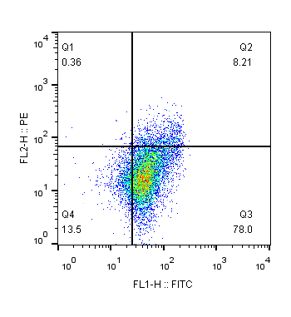


**siRNA parkin+prp**

**Figure 5**

**Figure 5D**


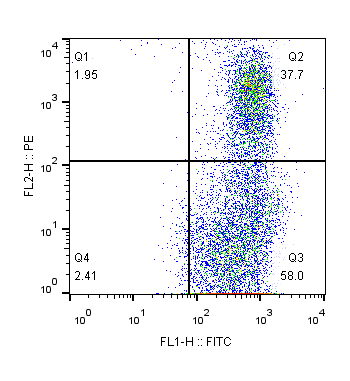


**Control**


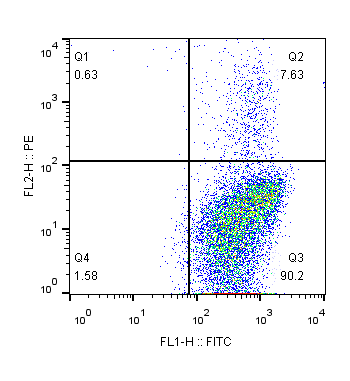


**PrP106-126 24h**


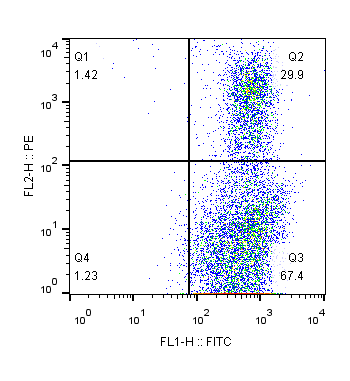


**Overexpress parkin**


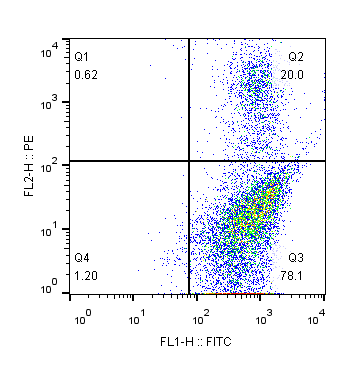


**Overexpress parkin+prp106-126**


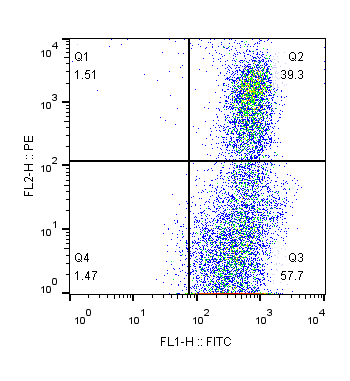


**NMN**


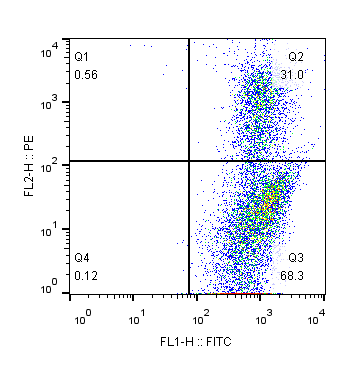


**NMN+prp106-126**


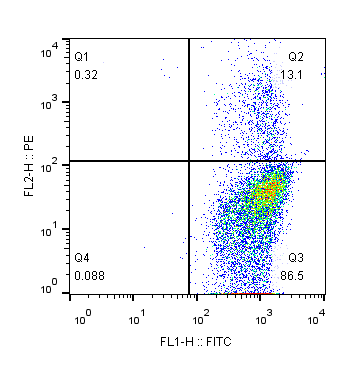


**UA**


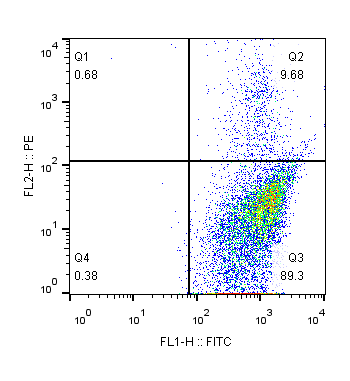


**UA+PrP106-126**

**Figure 5E**


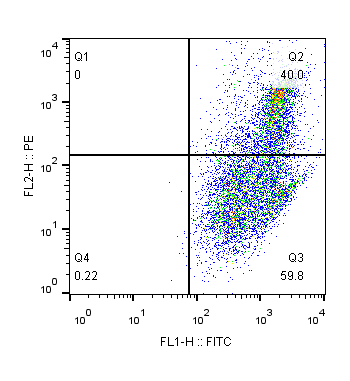


**Control**


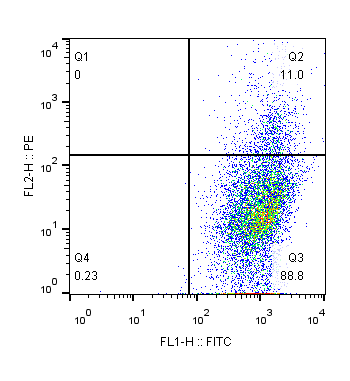


**PrP106-126 24h**


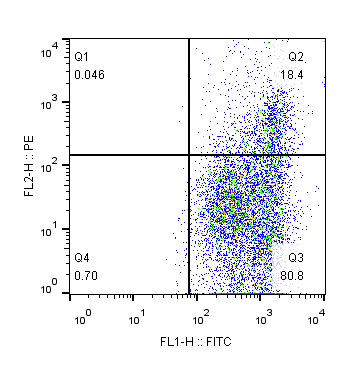


**siRNA parkin**


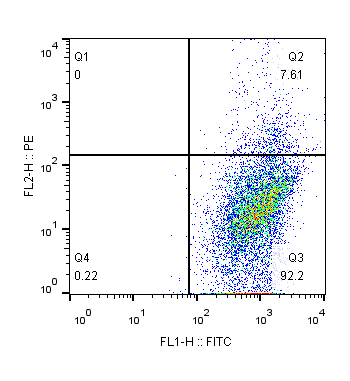


**siRNA parkin+prp106-126**
