# Supplementary material for: PINK1-parkin-mediated neuronal mitophagy deficiency in prion disease
Source: Cell Death Dis. 2022 Feb 18;13(2):162. doi: 10.1038/s41419-022-04613-2 (PMC8858315; doi:10.1038/s41419-022-04613-2)
Supplement: Supplementary file 5 — payment information [file 41419_2022_4613_MOESM5_ESM.docx]

Payment information：

Remitter’s Name: CHINA AGRICULTURAL UNIVERSITY

Address: NO.2 YUANMINGYUAN WEST ROAD, HAIDIAN DISTRICT, BEIJING, CHINA

Account No./Credit Card No. : 336356022629
