# Supplementary material for: PINK1-parkin-mediated neuronal mitophagy deficiency in prion disease
Source: Cell Death Dis. 2022 Feb 18;13(2):162. doi: 10.1038/s41419-022-04613-2 (PMC8858315; doi:10.1038/s41419-022-04613-2)
Supplement: Supplementary file 6 — third-party declarations [file 41419_2022_4613_MOESM6_ESM.docx]

third-party declarations:

all required third party permissions have been obtained.
